# Epidemiologic studies of modifiable factors associated with cognition and dementia: systematic review and meta-analysis

**DOI:** 10.1186/1471-2458-14-643

**Published:** 2014-06-24

**Authors:** May A Beydoun, Hind A Beydoun, Alyssa A Gamaldo, Alison Teel, Alan B Zonderman, Youfa Wang

**Affiliations:** 1 Laboratory of Epidemiology and Population Sciences, National Institute on Aging, NIA/NIH/IRP, 251 Bayview Blvd., Suite 100, Room #: 04B118, Baltimore, MD 21224, USA; 2 Graduate program in Public Health, Eastern Virginia Medical School, Norfolk, VA, UK; 3 Department of Epidemiology and Environmental Health, School of Public Health and Health Professions, University at Buffalo, State University of New York, New York, USA; 4 John Hopkins Global Center on Childhood Obesity, Where Systems Science Meets Public Health, Department of International Health, Johns Hopkins Bloomberg School of Public Health, Baltimore, MD, USA

**Keywords:** Cognition, Dementia, Alzheimer’s disease, Risk factor, Nutrition, Meta-analysis

## Abstract

**Background:**

Cognitive impairment, including dementia, is a major health concern with the increasing aging population. Preventive measures to delay cognitive decline are of utmost importance. Alzheimer’s disease (AD) is the most frequent cause of dementia, increasing in prevalence from <1% below the age of 60 years to >40% above 85 years of age.

**Methods:**

We systematically reviewed selected modifiable factors such as education, smoking, alcohol, physical activity, caffeine, antioxidants, homocysteine (Hcy), *n*-3 fatty acids that were studied in relation to various cognitive health outcomes, including incident AD. We searched MEDLINE for published literature (January 1990 through October 2012), including cross-sectional and cohort studies (sample sizes > 300). Analyses compared study finding consistency across factors, study designs and study-level characteristics. Selecting studies of incident AD, our meta-analysis estimated pooled risk ratios (RR), population attributable risk percent (PAR%) and assessed publication bias.

**Results:**

In total, 247 studies were retrieved for systematic review. Consistency analysis for each risk factor suggested positive findings ranging from ~38.9% for caffeine to ~89% for physical activity. Education also had a significantly higher propensity for “a positive finding” compared to caffeine, smoking and antioxidant-related studies. Meta-analysis of 31 studies with incident AD yielded pooled RR for low education (RR = 1.99; 95% CI: 1.30-3.04), high Hcy (RR = 1.93; 95% CI: 1.50-2.49), and current/ever smoking status (RR = 1.37; 95% CI: 1.23-1.52) while indicating protective effects of higher physical activity and *n*-3 fatty acids. Estimated PAR% were particularly high for physical activity (PAR% = 31.9; 95% CI: 22.7-41.2) and smoking (PAR%=31.09%; 95% CI: 17.9-44.3). Overall, no significant publication bias was found.

**Conclusions:**

Higher Hcy levels, lower educational attainment, and decreased physical activity were particularly strong predictors of incident AD. Further studies are needed to support other potential modifiable protective factors, such as caffeine.

## Background

Cognitive function refers to those mental processes that are crucial for the conduct of the activities of daily living. Such mental processes include attention, short-term and long-term memory, reasoning, coordination of movement and planning of tasks [1]. The prevalence of brain disorders affecting cognition (such as stroke and dementia) increases steadily in a linear fashion with age [2]. Cognitive impairment is a major health concern affecting loss of independence in daily activities in old age. Thus, special attention should be devoted to its prevention [3].

Dementia is relatively frequent in the elderly population and was shown to affect about 6.4% of European subjects over the age of 65 years [4]. A review of 50 original articles published between 1989 and 2002 using international data showed that prevalence of dementia for the very old group (85 years and over) varied from 16.7% in China [5] to 43% in Germany [6]. This variability was also reflected within separate age groups among the very old, ranging from 9.6% to 32% for the 85–89 age category and from 41% to 58% for the 95+ age group. Incidence varied between 47 and 116.6 per 1000 and a separate meta-analytic study estimated the incidence in that age group (i.e. 85+) to be around 104 per 1000 [7,8].

Alzheimer’s disease (AD) is the most frequent cause of dementia, increasing in prevalence from less than 1% below the age of 60 years to more than 40% above 85 years of age. The initial phase is generally marked by a progressive deterioration of episodic memory. Other impairments may be entirely absent in the beginning or consist of mild disturbances in naming and executive function. When the process advances, impairment spreads to other aspects of memory and other domains of cognition. Despite lack of curative treatment, epidemiological evidence reveals important risk factors for sporadic AD, many of which are non-modifiable (e.g. ApoE *ϵ4*, age and sex). This highlights the importance for further evaluation of modifiable risk and preventive factors in that these potential factors may not only delay the onset of cognitive decline, but also can be easily treated. Aside from AD, less frequently occurring forms of dementia include vascular dementia (VaD), mixed dementia, dementia with Lewy bodies (DLB) and Parkinson’s disease with dementia (PD-D), diagnostic criteria for which are described in Table 1. The relative prevalence of AD and VaD and the mixed version of both remain debatable. Ott and colleagues [9] estimated that for the very old, the prevalence of AD was 26.8% while that of VaD was 4.4%. However, VaD appears to be more frequent than AD in certain Japanese and Chinese populations [10].

**Table 1:** Diagnostic criteria of Alzheimer’s Disease (AD), Vascular Dementia (VaD), Mixed dementia (MD) and other dementias

**Diagnosis**	**Criteria**
*Alzheimer’s disease (AD)* (NINCDS-ADRDA) *Source*[11,12]:	Development of multiple cognitive deficits, with both memory impairment and one (or more) of the following cognitive disturbances:
	Aphasia (language disturbance)
	Apraxia (learned motor skills disturbance)
	Agnosia (visuospatial/sensory disturbance)
	Executive functioning (foresight, planning, insight anticipation)
	Significant impairment in social or occupational functioning, representing a significant decline from a previous level of functioning
	Other diagnostic criteria: Hachinski Ischemic Score, ICD-10; DSM-IV; ADDTC; updated NINCDS-ADRDA
*Vascular Dementia (VaD)* (NINDS-AIREN) *Source*[13]:	Cognitive decline from previous higher level of function in three areas of function including memory.
	Evidence of cerebrovascular disease by examination
	Evidence of cerebrovascular disease by neuroimaging
	Onset either abrupt or within three months of a recognized stroke.
*Vascular Dementia (VaD)* (Modified Hachinski Ischemia Score: ≥4) *Source*[14]:	Two-point items
	Abrupt onset
	History of stroke
	Focal neurologic symptoms
	One-point items
	Stepwise deterioration
	Somatic complaints
	History of hypertension
	Emotional incontinence
	Other diagnostic criteria: ICD-10; DSM-IV
*Mixed Dementias (MDs)*	
Hachinski Ischemic score	Score based on clinical features: ≤4 = AD; ≥7 = VaD; intermediate score of 5 or 6 = MD.
ICD-10	Cases that met criteria for VaD and AD
DSM-IV	Cases with criteria for primary degenerative dementia of the Alzheimer type and clinical or neuroimagery feature of VaD.
ADDTC	Presence of ischemic vascular disease and a second systemic or brain disorder.
NINDS-AIREN	Typical AD associated with clinical and radiological evidence of stroke.
*Other Dementias*	
*Fronto-Parietal Dementia (FTD) Source*[15]:	Behavioral or cognitive deficits manifested by either **(1)** or **(2)**:
	**(1)** Early and progressive personality change, with problems in modulating behavior; inappropriate responses/activities.
	**(2)** Early and progressive language changes, with problems in language expression, word meaning, severe dysnomia.
	Deficits represent a decline from baseline and cause significant impairment in social and occupational functioning.
	Course characterized by gradual onset and continuing decline in function.
	Other causes (eg, stroke, delirium) are excluded
	Gradual onset and progressive cognitive decline.
*Dementia with Lewy Bodies (DLB)* (Consensus Guidelines for the Clinical Diagnosis for Dementia with Lewy Bodies) *Source*[16]:	Fluctuating in cognitive performance: Marked variation in cognition or function, or episodic confusion/decreased responsiveness.
	Visual hallucinations: Usually well formed, unprovoked, benign.
	Parkinsonism: Can be identical to Parkinson’s Disease (PD), milder or symmetric.
*Parkinson’s Disease with Dementia (PD-D) Source*[17]:	Bradyphrenia (slowness of thought)
	Executive impairment
	Neuropsychiatric symptoms
	Dysphonia

*Sources*[17,18]:.

***Abbreviations: *** **ADDTC:** Alzheimer's Disease Diagnostic and Treatment Centers; **DSM-IV**: Diagnostic and Statistical Manual, 4^th^ edition; **ICD-10**: International Classification of Disease, 10^th^ edition; **NINCDS-ADRDA**: National Institute of Neurological and Communicative Disorders and Stroke -- the Alzheimer's Disease and Related Disorders Association; **NINDS-AIREN**: National Institute of Neurological and Communicative Disorders and Stroke--Association Internationale pour la Recherche et l’Enseignement en Neurosciences; **PD-D**: Parkinson’s disease with dementia.

The present review focuses on selected modifiable risk and protective factors of cognitive impairment, cognitive decline and dementia (including AD), given that they are commonly studied and provide reliable and comparable data. In particular, we focused on the risk and protective factors that could be grouped under three broad categories, namely socioeconomic, behavioral, and nutritional. Consequently, other known risk factors that are modifiable but did not fall under these categories were excluded (e.g. obesity, type 2 diabetes, hypertension, depression, traumatic head injury etc.). In addition, the latter risk factors are potential mediating factors in the causal pathway between our selected factors and cognitive outcomes (e.g. obesity, depression, type 2 diabetes and hypertension may be on the causal pathway between physical activity and cognition; the same for diet→depression, obesity, type 2 diabetes, hypertension→cognition) and thus are more appropriately studied together rather than with their antecedent putative causes. This is the first study to systematically review those selected modifiable risk and protective factors for cognitive health outcomes in cross-sectional and cohort studies while comparing the consistency of association between those factors and across study-level characteristics. It is also among few recent studies to compare the strength of association across those factors in relation to incident AD using a similar approach [19,20]. Our findings could help guide future research and interventions.

## Methods

### Literature search

Using MEDLINE, we conducted a systematic review of the literature on cognitive function, decline and dementia focusing on specific risk factors. We considered both original research published between January 1990 and October 2012. We did not include titles prior to 1990 to ensure that diagnostic criteria for dementia and AD were comparable across studies. After an initial search using MESH keywords for risk factors (i.e. education, smoking, physical activity, caffeine/coffee/tea, alcohol, antioxidant/vitamin E, homocysteine and fatty acid) and a title containing the words (cognitive, dementia and Alzheimer), we assessed the retrieved papers for relevance by reading the titles and abstracts. Among those that were selected for review, information was retrieved including study design, contextual setting, sample size, main outcome and key findings.

**Figure 1:**
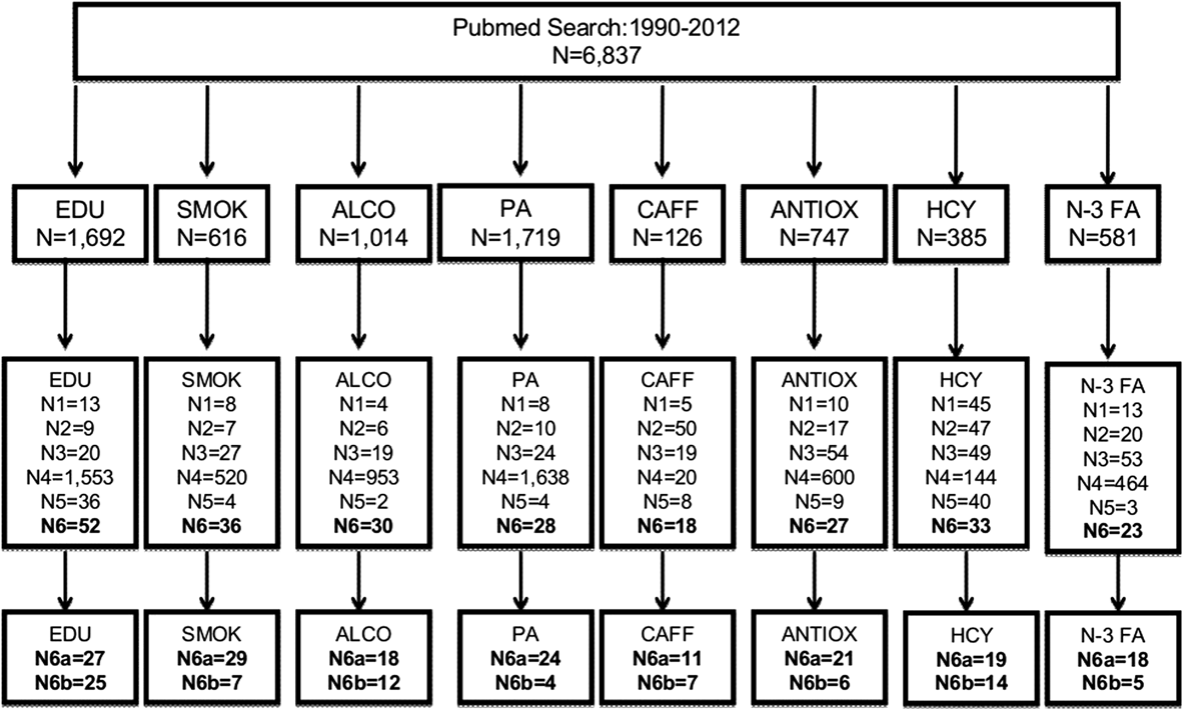
**Flowchart of study selection for systematic review and meta-analysis. ***Notes*: MEDLINE searches (1990–2012) included the following: (1) “Risk factor” as MESH term AND “Dementia” in title; (2) “Risk factor” as MESH term AND “Alzheimer” in title; (3) “Risk factor” as MESH term AND “Alzheimer” in title; (4) “Risk factor” as MESH term AND “cognitive” in title; (5) “Risk factor” in title and “cognitive” in title. Given that each search is not mutually exclusive of other searches, there were duplicates which were deleted from the final number of included studies. The following notations are defined follows: N1 = Studies excluded from all searches combined due to small sample size; N2 = Studies excluded from all searches combined due to design being neither cross-sectional nor cohort; N3 = Studies excluded from all searches combined due to being a review or a letter to the editor; N4 = Studies excluded from all searches combined due to lack of relevance to topic or hypothesis; N5 = Studies excluded from all searches combined for other reasons (e.g. special group of people); N6 = Final included studies; N6a = Final included cohort studies; N6b = Final included cross-sectional studies.

Final inclusion criteria were: **(1)** Study sample size > 300. Although this number is arbitrary, it was based on the fact that some study outcomes were relatively rare (e.g. incident AD: <10%) and thus a smaller sample size for a cohort study for example might yield an underpowered study, depending on the distribution of the risk factor. **(2)** Study design is either cross-sectional or cohort study (thus case-control studies, review articles, commentaries, and basic science papers were excluded); **(3)** Outcomes include dementia, AD, cognitive function, cognitive decline or cognitive impairment (including MCI)). Although all types of dementia were presented in our description of selected studies, focus was on the more prevalent sub-types including AD and VaD **(4)** Baseline sample includes general healthy population rather than special groups at risk (e.g. coronary heart disease patients). For the “cognitive decline” and “cognitive function” outcomes, we searched risk factors in the title to expand the range of studies selected beyond those based just on MESH keywords (i.e. “caffeine,” “alcohol” and all other risk factors were also searched in titles when the outcome was “cognitive”). Both cohort and cross-sectional studies that were selected are presented in Table 2. The MEDLINE search and the studies excluded are laid out in Figure 1, showing main reasons for exclusion and final number of studies included for each risk factor. After inclusion of a study, we did not examine cross-references in order to ensure the comparability of the search strategy between risk factors.

**Table 2:** Summary of epidemiologic studies of risk and protective factors for cognitive outcomes included in the review

**Study**	**Age/gender**	**Year**	**Country**	**Study design**	**Sample size**	**Outcomes**	**Findings**
*(1) Education*	*Hypothesis: Lower education is associated with lower cognitive function or higher rate of cognitive decline or increased risk of dementia(including AD)*						
[21]	65+/B	1990	China	Cross-sectional	N = 5,055	Prevalent AD and dementia	+
[22]	Mean:58.5/B	1991	Nigeria	Cross-sectional	N = 1,350	Cognitive function	+
[23]	65+/B	1992	France	Cross-sectional	N = 2,792	Cognitive function	+
[24]	68-77/B	1992	Finland	Cross-sectional	N = 403	Cognitive function	+
[25]	65+/B	1993	US	Cohort	N = 4,485	Cognitive decline	+
[26]	75+/B	1994	England	Cohort	N = 1,195	Indicent dementia	0
[27]	65+/B	1994	US	Cohort	N = 10,294	Incident cognitive impairment	+
[28]	55+/B	1995	US	Cohort	N = 3,330	Incident AD and VaD	+(VaD)
[29]	18+/B	1995	US	Cohort	N = 14,883	Indicent dementia	+
[9]	55-106/B	1995	The Netherlands	Cross-sectional	N = 7,528	Prevalent dementia, AD and VaD	+
[30]	65+/B	1996	US	Cross-sectional	N = 2,212	Prevalent dementia and cognitive impairment	+
[31]	68-78/B	1996	Finland	Cross-sectional	N = 403	Cognitive decline	+
[32]	70+/B	1997	Australia	Cohort	N = 652	Cognitive decline	+
[33]	65+/B	1997	US	Cohort	N = 642	Incident AD	+
[34]	50-80/B	1997	Austria	Cross-sectional	N = 1,927	Cognitive function	+
[35]	Mean:75/M	1997	The Netherlands	Cohort	N = 528	Cognitive decline	+
[36]	69-74/M	1997	Sweden	Cross-sectional	N = 504	Cognitive function	+
[37]	55-84/B	1997	The Netherlands	Cohort	N = 5,825	Cognitive function, decline and incident/prevalent dementia	+
[38]	65-84/B	1997	The Netherlands	Cohort	N = 2,063	Incident dementia	+(IQ better predcitor)
[39]	47-68/B	1998	US	Cross-sectional	N = 14,000	Cognitive function	+
[40]	60+/B	1998	Italy	Cross-sectional	N = 495	Prevalent dementia	0
[41]	65+/B	1998	Taiwan	Cross-sectional	N = 2,915	Prevalent dementia, AD and VaD	+(AD)
[42]	18+/B	1999	US	Cohort	N = 1,488	Cognitive decline	+
[43]	65+/B	1999	France	Cohort	N = 3,675	Incident AD	+
[44]	55-106/B	1999	The Netherlands	Cohort	N = 6,827	Incident dementia	+(women)
[45]	85+/B	2000	Sweden	Cohort	N = 494	Cognitive decline and function	+
[46]	75+/B	2001	Sweden	Cohort	N = 1,296	Incident dementia and AD	+
[47]	65+/B	2002	Spain	Cohort	N = 557	Cognitive decline	+
[48]	70+/B	2002	US	Cross-sectional	N = 6,577	Cognitive function	+
[49]	65+/B	2002	Brazil	Cross-sectional	N = 1,656	Prevalent dementia and AD	+
[50]	65+/B	2002	Italy	Cross-sectional	N = 1,016	Prevalent AD and VaD	+
[51]	45-59/M	2002	US	Cross-sectional	N = 1,839	Cognitive function	+
[52]	70-79/W	2003	US	Cohort	N = 19,319	Cognitive function and decline	+
[53]	70-79/B	2005	US	Cohort	N = 4,030	Cognitive decline	+(ApoE-)
[54]	66+/W	2006	US	Cohort	N = 6,314	Cognitive function and decline	+
[55]	Mean age: ~75/B	2006	US	Cohort	N = 2,786	Incident dementia	+(both whites and blacks)
[56]	55+/B	2006	China	Cross-sectional	N = 34,807	Prevalent AD and VaD	+(AD)
[57]	50+/B	2006	China	Cross-sectional	N = 16,095	Prevalent dementia and AD	+
[58]	65+/B	2007	Guam	Cross-sectional	N = 2,789	Prevalent dementia and AD	+
[59]	64-81/B	2007	The Netherlands	Cross-sectional	N = 578	Cognitive function	+
[60]	60-64/B	2009	Australia	Cohort	N = 416	Cognitive decline	0
[61]	30-64/B	2009	US	Cross-sectional	N = 1,345	Cognitive function	+(literacy better predictor)
[62]	65-96/B	2009	Spain	Cross-sectional	N = 1,074	Prevalent dementia	+
[63]	80+/B	2009	UK	Cohort	N = 3,336	Incident dementia	+
[64]	Mean:72/B	2009	US	Cohort	N = 6,000	Cognitive function and decline	+(cognitive function) 0(cognitive decline)
[65]	60+/B	2010	Malaysa	Cross-sectional	N = 2,980	Prevalent dementia	+
[66]	55+/B	2010	India	Cross-sectional	N = 2,466	Prevalent dementia and AD	+
[67]	65+/B	2010	Brazil	Cross-sectional	N = 2,003	Cognitive function	+
[68]	60+/B	2011	Brazil	Cohort	N = 1,461	Cognitive decline	-
[69]	60-98/B	2011	Italy	Cohort	N = 1,270	Incident cognitive impairment	+
[70]	60+/B	2011	Mexico	Cohort	N = 7,000	Prevalent and incident dementia	+
[71]	54-95/B	2011	US	Cohort	N = 1,014	Cognitive decline	0
Study	Age/gender	Year	Country	Design	Sample size	Outcome	Finding
*(2) Behavioral*							
*(2.1.) Smoking*	*Hypothesis: Current or ever smoking status is associated with lower cognitive function or higher rate of cognitive decline or increased risk of dementia(including AD)*						
[72]	65+/B	1993	US	Cohort	N = 1,201	Cognitive decline	0
[73]	65+/B	1994	France	Cross-sectional	N = 3,770	Prevalent AD, cognitive impairment	0
[74]	74+/B	1996	US	Cohort	N = 647	Cognitive function	0
[75]	Mean:58.6/M	1997	US	Cohort	N = 3,429	Cognitive impairment	+
[36]	69-74/M	1997	Sweden	cross-sectional	N = 504	Cognitive function	+
[76]	75+/B	1998	Australia	Cohort	N = 327	Incident dementia and AD	0
[77]	adults/B	1998	US	Cohort	N = 1,469	Cognitive function	0
[78]	55+/B	1998	US	Cohort	N = 6,870	Incident dementia and AD	+(ApoE4^−^)
[79]	56-69/M	1999	US	Cross-sectional	N = 569	Cognitive impairment	+(ApoE4^−^)
[80]	45-59/M	1999	UK	Cross-sectional	N = 1,870	Cognitive function	0
[81]	65+/B	2000	UK	Cohort	N = 889	Cognitive Impairment	+
[82]	Mean: 81/M	2000	UK	Cohort	N = 34,439	Definite or probable AD	0
[83]	45-70/B	2002	Netherlands	Cohort	N = 1,927	Cognitive change	+
[84]	65+/B	2003	Taiwan	Cohort	N = 798	Cognitive decline	0
[85]	43-53/B	2003	UK	Cohort	N = 3,035	Cognitive decline	+
[86]	Mean:78/M	2003	US	Cohort	N = 3,734	Incident AD	+
[87]	60+/B	2003	China	Cross-sectional	N = 3,012	Cognitive impairment	+
[88]	60+/B	2004	China	Cohort	N = 2,820	Incident dementia and AD	+
[89]	65+/B	2004	European cohorts	Cohort	N = 17,610	Cognitive decline	+
[90]	65-84/B	2004	Italy	Cohort	N = 5,632	Mild cognitive impairment	0
[91]	40-80/M	2004	The Netherlands	Cross-sectional	N = 900	Cognitive function	0
[92]	Mean:75/B	2005	US	Cohort	N = 791	Cognitive function and decline	+(75 + and ApoE4^−^)
[93]	40-44/B	2005	US	Cohort	N = 8,845	Incident dementia	+
[94]	50+/B	2006	UK	Cohort	N = 2,000	Cognitive function	+
[95]	55+/B	2007	The Netherlands	Cohort	N = 6,868	Incident dementia and AD	+
[96]	43-70/B	2008	The Netherlands	Cohort	N = 1,964	Cognitive decline	+
[97]	35-55/B	2008	France	Cohort	N = 4,659	Cognitive function	+(memory)
[98]	46-70/B	2009	US	Cohort	N = 11,151	Incident dementia	+
[99]	65+/B	2009	US	Cohort	N = 1,557	Cognitive decline	+
[100]	90-108/B	2009	China	Cross-sectional	N = 681	Cognitive impairment	+(men)
[63]	Mean:83.5/B	2009	UK	Cohort	N = 3,336	Incident dementia	0
[101]	65-79/B	2010	Finland	Cohort	N = 1,449	Incident dementia and AD	+
[102]	Mean:71.8/B	2010	Taiwan	Cohort	N = 1,436	Incident cognitive impairment	-
[103]	50y/M	2011	Sweden	Cohort	N = 2,268	Incident dementia and AD	+(non-AD)
[104]	Mean:60.1/B	2011	Finland	Cohort	N = 21,123	Incident dementia and AD	+
[105]	44-69/B	2012	UK	Cohort	N = 7,236	Cognitive decline	+(men)
Study	Age/gender	Year	Country	Design	Sample size	Outcome	Finding
*(2.2.) Alcohol*	*Hypothesis: Moderate alcohol consumption is protective against poorer cognitive function, higher rate of cognitive decline and dementia*						
[106]	65+/B	1996	US	Cross-sectional	N = 2,040	Cognitive function	+(J-shaped)
[107]	59-71/B	1997	France	Cross-sectional	N = 1,389	Cognitive function	+ (women)
[76]	75+/B	1998	Australia	Cohort	N = 327	Incident dementia and AD	0
[77]	40-80/B	1998	US	Cohort	N = 1469	Cognitive function	0
[108]	55-88/B	1999	USA	Cohort	N = 1786	Cognitive function	+(U-shaped)
[109]	65+/B	2001	US	Cross-sectional	N = 1,836	Cognitive function	+(U-shaped for men, linear for women)
[110]	Mean:70/B	2001	Italy	Cross-sectional	N = 15,807	Cognitive impairment	+(U-shaped)
[83]	45-70/B	2002	Netherlands	Cohort	N = 1,927	Cognitive change	+(women > men) (J-shaped)
[111]	18+/B	2000	US	Cohort	N = 1,448	Cognitive decline	+(women > men) (U-shaped)
[112]	53/B	2003	US	Cross-sectional	N = 10,317	Cognitive function	0
[87]	60+/B	2003	China	Cohort	N = 3,012	Cognitive impairment	-
[113]	65-79/B	2004	Finland	Cohort	N = 1,464	Cognitive function	+(U-shaped) - (ApoE4^+^)
[114]	65+/B	2004	US	Cohort	N = 4,417	Cognitive function	+(current drinker *vs*. former or abstainer)
[115]	35-55/B	2004	UK	Cohort	N = 10,308	Cognitive function	+(linear, some cognitive domains)
[116]	65+/B	2005	US	cohort	N = 1,624	Cognitive function	+(current drinker *vs*. former or abstainer)
[117]	Mean:74/B	2005	US	Cohort	N = 1,098	Cognitive function and decline	+(J-shaped)
[118]	43-53/B	2005	UK	Cohort	N = 1,764	Cognitive decline	Linear + (slower memory decline: men) -(faster psychomotor speed decline: women)
[119]	20-24,40-44,60-64/B		Australia	Cross-sectional	N = 7,485	Cognitive function	J-shaped + (light drinkers vs. abstainers)
[120]	70-81/W	2005	US	Cohort	N = 11,102	Cognitive function and decline	+(J-shaped) (cognitive decline)
[121]	65-89/M	2006	US	Cross-sectional	N = 760	Cognitive function	+(linear, J-shaped)
[122]	40+/B	2006	US	Cohort	N = 1,428	Cognitive decline	+(linear)
[123]	65-79/B	2006	Finland	Cross-sectional	N = 1,341	Cognitive function	+(linear)
[124]	65-84/B	2007	US	Cohort	N = 1,445	Incident MCI and MCI→ dementia	+(U-shaped)
[125]	50+/B	2010	China	Cohort	N = 30,499	MCI→ dementia	+(J-shaped)
[126]	50+/B	2010	China	Cross-sectional	N = 9,571-28,537	Cognitive function	+(occasional alcohol use vs. none)
[127]	65+/B	2009	China	Cross-sectional	N = 314	Cognitive impairment	+(U-shaped)
[128]	70/B	2011	UK	Cross-sectional	N = 922	Cognitive function	+(linear, verbal memory)
[129]	55+/B	2011	US	Cohort	N = 1,337	Cognitive function	0 -(executive function)
[130]	55+/B	2011	France	Cross-sectional	N = 4,073	Cognitive function	-(high alcohol use, Low SES)
[131]	45+/B	2012	US	Cohort	N = 571	Cognitive decline	+(heavy drinking)
Study	Age/gender	Year	Country	Design	Sample size	Outcome	Finding
*(2.3.) Physical activity*	*Hypothesis: Physical activity is protective against poorer cognitive function, higher rate of cognitive decline and dementia(including AD)*						
[132]	70+/B	2001	Hong Kong	Cohort	N = 2030	Cognitive impairment	+
[133]	65+/B	2001	Canada	Cohort	N = 4615	Incident cognitive impairment and AD	+
[134]	65-84/M	2001	Netherlands	Cohort	N = 347	Cognitive decline	+(ApoE4+)
[135]	65+/F	2001	US	Cohort	N = 5,925	Cognitive decline	+
[136]	75+/B	2003	US	Cohort	N = 469	Incident dementias (AD, VaD and others)	+
[137]	71-93/M	2004	US	Cohort	N = 2257	Incident dementia and AD	+
[138]	65+/B	2004	US	Cohort	N = 1146	Cognitive decline	+
[139]	80+/M	2004	European countries	Cohort	N = 295	Cognitive decline	+
[140]	70-81/W	2004	US	Cohort	N = 18766	Cognitive decline	+
[141]	65+/M	2005	US	Cohort	N = 3375	Incident dementia and AD	+(ApoE4-)
[142]	65-79/B	2005	Sweden	Cohort	N = 1449	Incident dementia and AD	+
[143]	65+/B	2005	US	Cohort	N = 4055	Cognitive decline	-
[144]	75+/B	2006	Sweden	Cohort	N = 776	Incident dementia	+
[145]	65+/B	2006	US	Cohort	N = 1740	Incident dementia and AD	+
[146]	65+/W	2010	US	Cross-sectional	N = 9344	Cognitive impairment	+
[147]	60+/B	2008	Greece	Cohort	N = 732	Cognitive impairment	+
[148]	71-92/M	2008	US	Cohort	N = 2263	Dementia	+
[149]	70+/B	2009	Italy	Cross-sectional	N = 668	Cognitive decline	+
[100]	90-108/B	2009	China	Cross-sectional	N = 681	Cognitive impairment	+
[150]	65+/B	2009	US	Cohort	N = 1880	Incident AD	+
[151]	70-79/B	2009	US	Cohort	N = 2509	Cognitive function and decline	+
[152]	Mean:51y/B	2010	Iceland	Cohort	N = 4945	Cognitive function and dementia	+
[153]	55+/B	2010	Germany	Cohort	N = 3903	Incident cognitive impairment	+
[154]	60+/B	2010	US	Cohort	N = 5903	Cognitive function	+
[155]	65+/W	2010	US	Cross-sectional	N = 9344	Cognitive function and impairment	+
[156]	Mean:82/B	2012	US	Cohort	N = 716	AD Cognitive decline	+
[157]	40-84/B	2012	US	Cohort	N = 405 (40–59 years) N = 342 (60–84 years)	Cognitive function	+
[158]	65+/B	2012	US	Cohort	N = 2491	Incident dementia & AD	+
Study	Age/gender	Year	Country	Design	Sample size	Outcome	Finding
*(3) Nutritional*							
*(3.1) Caffeine(coffee or tea)*	*Hypothesis: Caffeine consumption is protective against poorer cognitive function, higher rate of cognitive decline and dementia*						
[159]	18+/B	1993	UK	Cross-sectional	N = 9,003	Cognitive function	+(caffeine)
[160]	Mean: 73/B	2002	US	Cross-sectional	N = 1,528	Cognitive function	0(coffee)
[161]	24-81/B	2003	The Netherlands	Cohort	N = 1,376	Cognitive change	0(caffeine)
[162]	70+/B	2006	Japan	Cross-sectional	N = 1,003	Cognitive impairment	+(green tea)
[163]	Mean ~ 75/M	2007	Finland, the Netherlands and Italy	Cohort	N = 667	Cognitive decline	+(coffee, J-shaped)
[164]	55+/B	2008	Singapore	Cohort	N = 1,438	Cognitive impairment and decline	+(tea)
[165]	65-79/B	2009	Finland	Cohort	N = 1,409	Incident dementia and AD	+(coffee), 0(tea)
[100]	90+/B	2009	China	Cross-sectional	N = 681	Cognitive impairment	+(tea, men)
[166]	65+/B	2009	Finland	Cohort	N = 2,606	Cognitive function, incident dementia and MCI	0(coffee)
[167]	70-74/B	2009	Norway	Cross-sectiona	N = 2,031	Cognitive impairment	+(tea)
[168]	17-92/B	2009	UK	Cross-sectional	N = 3,223	Cognitive function	0(caffeine)
[169]	70/B	2010	UK	Cohort	N = 923	Cognitive function	+(coffee); −(tea)
[170]	55+/B	2010	Singapore	Cross-sectional	N = 716	Cognitive function	+(tea)
[171]	65+/B	2010	France	Cohort	N = 641	Cognitive decline	+(caffeine, women)
[172]	65+/B	2010	Portugal	Cohort	N = 648	Cognitive decline	+(caffeine, women)
[173]	65+/B	2011	US	Cohort	N = 4,809	Cognitive decline	+(caffeine, women)
[174]	Mean:54/M	2011	US	Cohort	N = 3,494	Incident dementia and cognitive impairment	0(caffeine)
[175]	Mean:91.4/B	2012	Singapore	Cohort	N = 7,139	Cognitive change	+(tea)
Study	Age/gender	Year	Country	Design	Sample size	Outcome	Finding
*(3.2) Antioxidants/Vitamin E*	*Hypothesis: Antioxidants, including vitamin E, are protective against poorer cognitive function, higher rate of cognitive decline and dementia(including AD)*						
[176]	55-95/B	1996	Netherlands	cohort	N = 5,182	Cognitive function	+
[177]	66-97/B	1998	US	Cohort	N = 1,059	Cognitive function	0
[178]	65+/B	1998	US	Cohort	N = 633	Incident AD	+
[179]	5075/B	1998	Austria	Cross-sectional	N = 1,769	Cognitive performance	+(Vit. E)
[180]	71-93/M	2000	Hawaii	Cohort	N = 3,385	Incident AD, VaD, MD and OD	+(VaD)
[181]	48-67/B	2000	US	Cross-sectional	N = 12,187	Cognitive performance	0
[182]	55+/B	2002	Netherlands	Cohort	N = 5,395	Incident AD	+
[183]	65+/B	2002	US	Cohort	N = 815	Incident AD	+(Vit. E, ApoE4^−^)
[184]	65-102/B	2002	US	Cohort	N = 2,889	Cognitive decline	+
[185]	65+/B	2003	US	Cohort	N = 2,969	Incident dementia Incident AD	0
[186]	70-79/W	2003	US	Cohort	N = 14,968	Cognitive function	+(Vit. E)
[187]	65+/B	2003	US	Cohort	N = 980	Incident AD	0
[188]	45-68/M	2004	US	Cohort	N = 2,459	Incident dementia and AD	0
[189]	65+/B	2004	US	Cohort	N = 4,740	Incident and prevalent AD	+
[190]	65+/B	2005	Italy	Cross-sectional	N = 1,033	Prevalent dementia and cognitive impairment	+
[191]	55+/B	2005	Netherlands	Cross-sectional	N = 3,717	Prevalent AD	0
[192]	65-105/B	2005	US	Cohort	N = 616	Incident Dementia Incident AD	0
[193]	65+/B	2005	Canada	Cohort	N = 894	Cognitive decline Dementia	+
[194]	65+/B	2005	US	Cohort	N = 3,718	Incident AD Cognitive function	+
[195]	Mean:73.5/B	2007	France	Cross-sectional	N = 589	Cognitive function	+
[196]	60+/W	2007	US	Cohort	N = 526	Cognitive impairment	+(Vit. E)
[197]	65+/B	2007	US	Cohort	N = 3,831	Cognitive function	+
[198]	65+/B	2008	US	Cohort	N = 3,376	Cognitive function	+
[199]	65+/B	2008	US	Cohort	N = 2,969	Incident Dementia Incident AD	0
[200]	65+/B	2008	Italy	Cohort	N = 761	Cognitive impairment	+(Vit. E Sub-type)
[201]	70+/W	2010	US	Cohort	N = 16,010	Cognitive function & decline	+(cognitive function)
[202]	70/B	2011	UK	Cross-sectional	N = 882	Cognitive function	0
Study	Age/gender	Year	Country	Design	Sample size	Outcome	Finding
*(3.3) Homocysteine*	*Hypothesis: Homocysteine is a risk factor for poorer cognitive function, higher rate of cognitive decline and dementia (including AD)*						
[203]	55+/B	1999	Netherlands	Cohort	N = 702	Cognitive function and decline	0
[204]	60+/B	2002	UK	Cross-sectional	N = 391	Cognitive function	+
[205]	55+/B	2002	The Netherlands	Cross-sectional	N = 1,077	Cognitive function	+
[206]	Mean:76/B	2002	US	Cohort	N = 1,092	Incident AD	+
[207]	60+/B	2003	US	Cross-sectional	N = 1,789	Global cognitive function	+
[208]	Mean:73/B	2003	Italy	Cross-sectional	N = 650	Cognitive function	+
[209]	65+/B	2004	US	Cohort	N = 679	Incident and prevalent AD	0
[210]	Mean:72/B	2005	Turkey	Cohort	N = 1,249	Incident dementia, AD, MCI	0
[211]	60+/B	2005	US	Cross-sectional	N = 1,789	Cognitive impairment and dementia	0
[212]	40-82/B	2005	US	Cross-sectional	N = 2,096	Cognitive function	+(60 + y)
[213]	70-79/B	2005	US	Cohort	N = 499	Cognitive function and decline	+(cognitive function)
[214]	85+/B	2005	Netherlands	Cohort	N = 599	Cognitive impairment and decline	+(with impairment)
[215]	65+/B	2005	Switzerland	Cohort	N = 623	Incident MCI, dementia, AD and VaD	+
[216]	60+/B	2005	US	Cross-sectional	N = 1,789	Cognitive impairment and dementia	+
[217]	Mean:74/B	2005	Italy	Cohort	N = 816	Incident AD	+
[218]	50-70/B	2005	US	Cross-sectional	N = 1,140	Cognitive function	+
[219]	50-85/M	2005	US	Cohort	N = 321	Cognitive decline	+
[220]	Mean:62/B	2006	US	Cross-sectional	N = 812	Cognitive function	+
[221]	55+/B	2006	China	Cross-sectional	N = 451	Cognitive function	+
[222]	Mean:59/B	2006	The Netherlands	Cohort	N = 345	Cognitive function	+
[223]	65+/B	2007	UK	Cohort	N = 1,648	Cognitive decline	+
[224]	60-101/B	2007	US	Cohort	N = 1,779	Incident dementia and MCI	+
[225]	60-85/B	2007	South Korea	Cross-sectional	N = 1,215	Prevalent MCI	+
[226]	26-98/B	2008	US	Cross-sectional	N = 911	Cognitive function	+(ApoE4+)
[227]	65+/B	2008	Korea	Cross-sectional	N = 607	Cognitive function	+
[228]	Mean:72/B	2008	Korea	Cohort	N = 518	Incident dementia and AD	+
[229]	Mean:77/B	2009	US	Cohort	N = 516	Prevalent and incident MCI	0
[230]	38-85/B	2010	Sweden	Cohort	N = 488	Incident dementia	0
[231]	65+/B	2010	The Netherlands	Cohort	N = 1,076	Cognitive decline	+
[232]	Mean:78/W	2011	Germany	Cross-sectional	N = 420	Cognitive function	+
[233]	38-60/W	2011	Sweden	Cohort	N = 1,368	Incident dementia and AD	+
[234]	70-89/M	2012	Australia	Cohort	N = 4,227	Incident dementia	+
[235]	70-89/M	2012	Australia	Cohort	N = 1,778	Incident cognitive impairment	+
Study	Age/gender	Year	Country	Design	Sample size	Outcome	Finding
*(3.4) n-3 fatty acids*	*Hypothesis: n-3 fatty acids are protective against poorer cognitive function, higher rate of cognitive decline and dementia(including AD)*						
[236]	69-89/M	1997	Netherlands	cohort	N = 476	Cognitive impairment & decline	0
[237]	55+/B	1997	Netherlands	Cohort	N = 5,386	Incident dementia and AD	+
[238]	55+/B	2002	Netherlands	Cohort	N = 5,395	Incident dementia and AD	0
[239]	65-94/B	2003	US	Cohort	N = 815	Incident AD	+
[240]	45-70/B	2004	Netherlands	Cross-sectional	N = 1,613	Cognitive function	+
[241]	65+/B	2005	US	Cohort	N = 3,718	Cognitive decline	0
[242]	65+/B	2007	France	Cohort	N = 8,085	Incident dementia and AD	+(ApoE4-)
[243]	50+/B	2007	US	Cohort	N = 2,251	Cognitive decline	+(hypertensive, Dyslipidemic)
[244]	Mean:76/B	2007	Italy	Cross-sectional	N = 935	Prevalent dementia	+
[245]	50-70/B	2007	Netherlands	Cohort	N = 404-807	Cognitive function and change	+(change)
[246]	50+/B	2008	US	Cohort	N = 2,251-7,814	Cognitive decline	+(hypertensives)
[247]	65-80/B	2008	Finland	Cohort	N = 1,449	MCI and cognitive function	+
[248]	Mean:78/B	2008	France	Cohort	N = 1,214	Incident dementia	+
[249]	65+/B	2009	Multi-national	Cross-sectional	N = 14,960	Prevalent dementia	+
[250]	55+/B	2009	Netherlands	Cohort	N = 5,395	Incident dementia and AD	0
[251]	65+/B	2009	Canada	Cohort	N = 663	Incident dementia or AD	0
[252]	Mean:68/M	2009	Netherlands	Cohort	N = 1,025	Cognitive function	0
[253]	76-82/W	2009	France	Cohort	N = 4,809	Cognitive decline	+
[254]	Mean:75/B	2010	Spain	Cross-sectional	N = 304	Cognitive impairment	+
[255]	35-54/B	2010	US	Cross-sectional	N = 280	Cognitive function	+
[256]	55+/B	2011	Singapore	Cohort	N = 1,475	Cognitive function and decline	+(supplements)
[257]	Mean:~64/B	2011	France	Cohort	N = 3,294	Cognitive impairment	+
[258]	65+/B	2011	France	Cohort	N = 1,228	Cognitive decline	+(ApoE4^+^; depressed)

^+^Hypothesized association; − Association against hypothesis; 0: No association.

*Abbreviations*: **AD**: Alzheimer’s Disease; **ApoE**: Apolipoprotein E; **B**: Both; **M**: Men; **MCI**: Mild Cognitive Impairment; MD = Mixed Dementia; OD = Other dementia; **UK**: United Kingdom; **US**: United States; **VaD**: Vascular Dementia; **W**: Women.

Out of a total search of 6,837 titles and abstracts between 1990 and 2012 (range:126 for caffeine to 1,692 for education), 247 published original epidemiologic studies (167 cohort-, 80 cross-sectional studies) were included in our review. A database was built accordingly using Endnote ver. X3 [259]. Each study was summarized in Table 2 by listing the sample characteristics (age, gender, country), study design, sample size and type of outcome. Given the diversity of types of outcomes, a quantitative meta-analysis for all studies with all outcomes was not possible. Thus, a qualitative method to assess overall consistency was conducted. This analysis was mainly based on the hypothesized direction of association and the final conclusion of the study. Thus, main findings based on the pre-set hypothesis was coded (+: supports the hypothesis; 0: no significant finding; −: against the hypothesis). In addition, within +, we coded studies as partially supporting the hypothesis for three main reasons: “some outcomes but not others”, “some exposures but not others”, “some sub-group(s) but not others”. These papers are sorted by risk factor, year of publication and first author’s last name.

### Descriptive analysis

In the descriptive part of the analysis, a data point consisted of a study finding within a design/risk factor dyad (e.g. cohort/education). Using the data points, we conducted an analysis to assess consistency of positive findings across risk factors and study designs (cohort vs. cross-sectional). In particular, we estimated the % of positive findings for all participants and most outcomes; % of positive findings for some outcomes or exposures but not others; % of positive findings for sub-groups; % null findings; % of findings against hypothesized direction. In addition, study-level characteristics (e.g. year and country of publication, study design, type of cognitive outcome, sample size, age group, sex) were described in detail and compared across risk factors, using *χ*^2^ test, independent samples *t*-test and one-way ANOVA.

### Consistency analysis: all data points

In this part of the analysis, we modeled study finding as a binary outcome coded as 0=”null finding or finding against hypothesized direction” (referent category), 1=”positive or partially positive finding”, as a function of study-level characteristics using a logistic regression model. The study-level characteristics were entered as main effects as follows: **(1)** Year of publication; **(2)** Country of publication (1 = US, 2 = European country, 3 = Others), (3) risk factor (1 = education, 2 = smoking, 3 = alcohol, 4 = physical activity, 5 = caffeine, 6 = antioxidants, 7 = homocysteine, 8 = *n*-3 fatty acids); **(4)** sample size (when a range was provided, the average was taken), (5) Study participant age group: 1 = contains ages <65y, 0 = does not contain ages <65y; **(6)** Participant gender composition: 1 = Men only; 2 = Women only; 3 = Both; **(7)** Study design: 1 = cross-sectional; 2 = cohort; **(8)** Number of cognitive outcomes included in the study (e.g. 1 if only incident AD was the outcome; 2 if it is both incident AD and incident dementia); **(9)** General category of cognitive outcome(s): 1 = dementia/AD/impairment; 2 = cognitive function/decline; 3 = both.

### Meta-analysis: data points with incident AD and selected risk or protective factors

Focusing on data points with incident AD as an outcome, we conducted further meta-analysis to assess the strength of the association between selected risk or protective factors and this outcome. This analysis was thus restricted to prospective cohort studies with available data points that had comparable measurements for each risk/protective factor, thus allowing to estimate a pooled measure of association across those data points and studies. The original reported odd ratios (ORs), relative risks (RRs) or Hazard Ratios (HRs) were combined into a pooled value with 95% confidence interval (CI). The RRs were then pooled using *random effects* models when included study data points were deemed heterogeneous based on the Q-test for homogeneity (p < 0.05) or *fixed effect* when study data points were homogenous (p > 0.05), which are also presented among results. As such, a summary or pooled RR was provided using forest plots and computed by computing the weighted average of the natural logarithm of each relative measure of interest weighting by the inverse of each RR’s respective variance [260]. Random effects models that further incorporated between-study variability were conducted using DerSimonian and Laird’s methodology.

Considering estimates of exposure prevalence from the largest study with available data on each exposure, we also computed a population attributable risk percentage (PAR%) by pooling data points from all studies together.

(1.1) $$\begin{array}{l} \mathrm{PAR}{\%}_{p,\mathrm{lcl},\mathrm{ucl};\mathrm{ij}}=\frac{100\times \left({\mathrm{Pr}}_{\text{exp}}\times \left(RR_{p,\mathrm{lcl},\mathrm{ucl};\mathrm{ij}}-1\right)\right)}{1+({\mathrm{Pr}}_{\text{exp}}\left(RR_{p,\mathrm{lcl},\mathrm{ucl};\mathrm{ij}}-1\right)}\\ \;=\left(1-\theta_{\mathrm{ij}}\right)\times 100 \end{array}$$

(1.2) $$\begin{array}{l} \mathrm{Var}\left(\theta_{\mathrm{ij}}\right)=\mathrm{Var}(1-\theta_{\mathrm{ij}})=(1-\mathrm{PA}R_{p;\mathrm{ij}}){}^{2}\times \mathrm{Var}(\mathrm{Ln}(1-\mathrm{PA}R_{p;\mathrm{ij}}))\\ \;={\left(1-\mathrm{PA}R_{p;\mathrm{ij}}\right)}^{2}\times (\mathrm{Ln}(1-\mathrm{PA}R_{\mathrm{lcl};\mathrm{ij}})-\mathrm{Ln}(1-\mathrm{PA}R_{\mathrm{ucl};\mathrm{ij}})/3.92){}^{2} \end{array}$$

(1.3) $$\mathrm{PAR}{\%}_{95\%\mathrm{CI};\mathrm{ij}}=\mathrm{PAR}{\%}_{p,\mathrm{ij}}\pm 1.96\times \sqrt{\mathrm{Var}\left(\theta_{\mathrm{ij}}\right)}\times 100$$

As shown in Equations 1.1, 1.2 and 1.3, RR (point estimates per study and data point; 95% CI) was applied to the formula and Pr_exp_ was the estimated prevalence of each exposure. The estimation of SE for PAR% was obtained using the delta method [261].

Finally, in order to examine publication bias, we used Begg’s funnel plots; each RR point estimate was plotted against their corresponding standard errors (SE) for each study on a logarithmic scale [262,263], for all exposures combined. This type of bias was also formally tested using the Begg-adjusted rank correlation tests [264] and the Egger’s regression asymmetry test [265]. All analyses were conducted with STATA 11.0 (StataCorp, College Station, TX) [266]. Type I error was set at 0.05 for all measures of association.

## Results and discussion

### Socio-economic Status (SES) as indicated by education

Early life conditions are related to cognitive development and abilities in childhood and cognitive function in adulthood. Low educational attainment and other markers of low socio-economic position (SEP) were associated with poorer cognitive function in adulthood and age-related cognitive decline and impairment, as well as greater risk or prevalence of dementia and AD in the elderly. In this study, we focused our attention on education as a maker of SES, given that it is the most commonly studied protective factor.

Several possible mechanisms support the finding that less education is related to cognitive decline: First, education may exert direct effects on brain structure early in life by increasing synapse number or vascularization and creating cognitive reserve. This was named the “reserve capacity” hypothesis. Thus, this hypothesis states that early life conditions affect the pace of cognitive decline in later life [38]. Education in early life may have effects in later life if persons with more education continue searching for mental stimulation (“the use it or lose it” hypothesis), which may lead to beneficial neurochemical or structural alterations in the brain [267]. Indeed, in one study, recent mental stimulation was associated with improved cognitive functioning [268]. Alternatively, education may act through several “behavioral mediators” to improve health in general, and cognitive functioning in particular [267]. This hypothesis was confirmed by a study using the Framingham cohort which suggested that education was uniquely protective against VaD and not associated with AD [28]. This finding was explained by mediating effects of other risk factors of cognitive decline, including smoking and hypertension, which in turn can initiate cerebrovascular damage. However, Lee and colleagues [52] found evidence contrary to this hypothesis by showing a sustained strong association between education and cognitive functioning after adjustment for behavioral and health-related factors.

**Figure 2:**
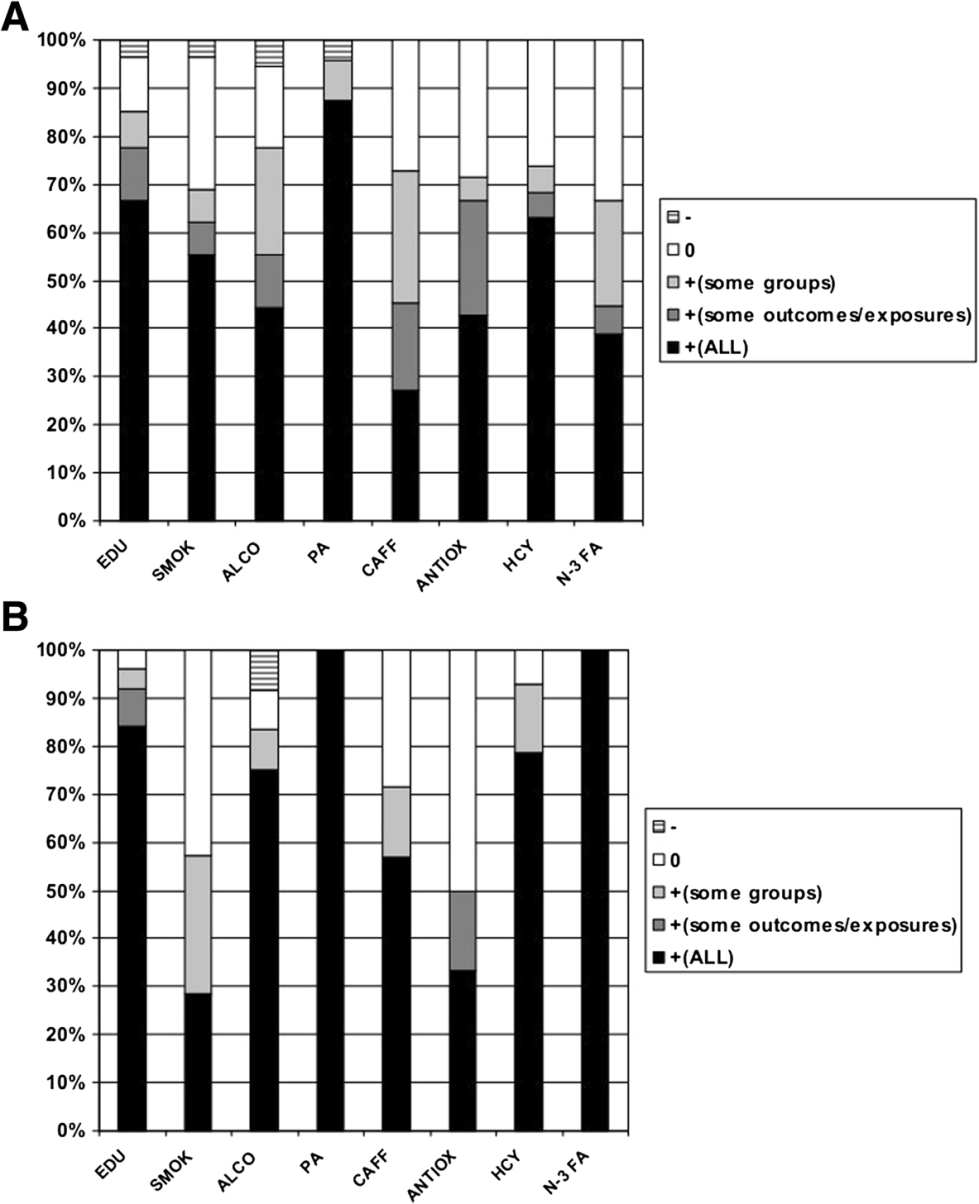
Main findings (%) of selected studies, given hypothesis: (A) Cohort Studies (B) Cross-sectional studies.

*Note:* +(ALL) = positive finding, given hypothesis, for all subjects and most outcomes of interest; +(some outcomes) = positive finding, given hypothesis, for all subjects and some outcomes of interest but not others; +(some groups) = positive finding, given hypothesis, for some groups and most outcomes of interest; 0 = null finding, given hypothesis; − = finding against hypothesized direction. *P-value based on *χ*^2^ test for independence between risk factor and finding.

Based on our findings (Table 2 and Figure 2A), 18 (66.7%) of the 27 cohort studies that met our selection criteria found that lower education was linked to a worse cognitive outcome in the overall population and for all studied outcomes, 1 found this relationship with incident VaD but not AD [28], 1 found the relationship with cognitive function but not decline [64], 1 concluded that IQ was a better predictor than education [38], and 2 detected a significant association in the hypothesized direction only in women [44] and in ApoE4- individuals [53]. The remaining four cohort studies did not find an association in the hypothesized direction [26,60,68,71].

The association between education and the studied cognitive outcomes was in the hypothesized direction, with higher education being protective, for the majority of the selected cross-sectional studies (21 out of 25, 84%), while 2 found an association with prevalent AD but not VaD [41,56], 1 found that literacy was a better predictor than education [61], and 1 failed to detect a significant association [40] (See Table 2 and Figure 2B).

### Behavioral factors

Several behavioral factors were selected, including smoking, alcohol drinking,and physical activity.

#### Smoking

Smoking is a risk factor for several chronic diseases, but its long-term relationship with dementia of various sub-types is still controversial. In fact, smoking is well known to increase the risk of stroke [269] and thus subsequent vascular types of dementia (VaD). However, many studies have concluded that smoking status influenced risk of VaD independently of stroke status and thus may have an effect beyond cerebrovascular disease. In addition, studies that have shown a direct impact of smoking on AD, suggest that smoking might in fact influence neurodegeneration. A vast amount of literature points to a role of smoking in oxidative stress and inflammation, both mechanisms believed to play a key role in AD [270].

However, it is also biologically plausible that smoking might protect against cognitive decline and AD, given that nicotine, a key active component of tobacco, may enhance the release of acetylcholine, increase the density of nicotnic receptors, therefore improving attention and information processing [271]. It is now known that AD is characterized by cholinergic system deficits which may be delayed through tobacco consumption [271,272].

Population-based evidence of an effect of smoking on cognitive outcomes was inconclusive, with most longitudinal studies reporting weak or null associations [63,72,74,76,77,82,84,90]. However, a number of other cohort studies have found a positive association between smoking and risk of incident dementia and AD [78,86,88,93,95,98,101,103,104] as well as incident cognitive impairment [75,81,90] and age-related cognitive decline [83,85,89,96,99,105].

For instance, the British 1946 birth cohort study pointed to the difficulty of finding an association between smoking and cognitive impairment given the differential high mortality of smokers especially among the elderly population [85]. After controlling for a range of socioeconomic and health status indicators (both physical and mental), they found that smokers who survive into later life may be at risk of clinically significant cognitive decline. However, these effects were accounted for largely by heavy smokers, i.e. those who smoked 20 cigarettes per day or more. Earlier research on middle aged adults suggested that current smoking and number of pack-years of smoking were related to reduced performance on tests of psychomotor speed and cognitive flexibility assessed approximately five years later [83]. Similar results were shown for cognitive decline in a large cohort study (Rotterdam study) conducted in multiple European countries [89] and in another more recent study conducted in the United States [92].

Among studies that examined incidence of AD in relation to smoking status, two of the largest European cohort studies reported conflicting results. While one found no relationship between smoking status and incident AD among a large sample of 34,439 older UK men (mean age: 81) [82], the recent 2011 study found that heavy smoking increased the risk of dementia and AD in a younger sample of 21,123 older Finnish adults (Mean age:60.1) that comprised both men and women [104].

In sum, 16 (55.2%) out of the 29 selected cohort studies linking smoking to the various cognitive outcomes found the relationship to be in the hypothesized direction in the entire population that was studied and for most outcomes of interest [75,81,83,85,86,88,89,92-96,98,99,101,104], while 2 found this relationship for some outcomes but not others [97,103] and 2 detected it for a sub-group of the total population [78,105], while the remaining 9 studies did not find an association [63,72,74,76,77,82,84,90] or found an association in the opposite direction [102]. (See Table 2 and Figure 2A).

Only 2 (28.6%) of the 7 cross-sectional studies found an association in the hypothesized direction [36,87], while 2 detected it for a sub-group of the total population [79,100], and the remaining 3 did not detect a significant association [73,80,91], (See Table 2 and Figure 2B).

#### Alcohol

Alcohol consumption in moderation was hypothesized to be protective against cognitive decline and impairment in old age. Several mechanisms may be involved in explaining the potential protective effect of moderate alcohol consumption on various cognitive outcomes. First, this effect might be mediated through cardiovascular risk factor reduction, partly through a dampening effect of ethanol on platelet aggregation, or through a modification of the serum lipid profile. Second, another potential mechanism in which alcohol can have a direct effect on cognitive function is through acetylcholine release in the hippocampus, which in turn enhances learning and memory [273].

The Rotterdam study [83] also examined the effect of alcohol use on cognition. They found that past alcohol consumption’s effect on speed and flexibility appeared to be slightly U-shaped, with the best performance observed among those who drank 1–4 glasses of alcohol per day, although this association was stronger among women than among men. Other studies also detected sex differences [106,107,111]. Light to moderate alcohol consumption was also found beneficial based on findings of other cohort and cross-sectional studies with a U- or J-shaped pattern observed [108-110,113,114,116,117,119-121,124-127]. However, in other studies, a linear dose–response relationship between alcohol use and improved cognition was noted, though the authors cautioned that these should not encourage increased alcohol consumption without an upper bound to this consumption [114,115,122,131].

In one cross-sectional study, a linear relationship between alcohol consumption and cognitive function was found in women but a U-shaped pattern was found in men [109]. One cohort study found that overall, moderate consumption was protective against poor cognitive function, but had an opposite relationship with cognitive function among ApoE4^+^ individuals [113], while another found that alcohol use in general was related to better cognition without effect modification by ApoE4 status [123]. Slower memory decline with increased alcohol consumption in men was found in one study, though the opposite relationship was found in the case of psychomotor speed among women [118]. The positive association between alcohol intake and memory was also noted in at least one other cross-sectional study for both men and women combined [128]. Moreover, heavy alcohol use was linked to poorer cognitive outcomes in a few studies [87,127,129,130]. Finally, only a few studies among those that were selected found no associations between alcohol consumption and cognitive outcomes [76,77,112].

In fact, 8 out of the 18 selected cohort studies (44%) linking alcohol consumption to the various cognitive outcomes, found the relationship to be in the hypothesized direction (but were U-shaped, J-shaped or linear) in the entire population that was studied and for most outcomes of interest [108,114,116,117,122,124,125,131], while 2 found this relationship for some outcomes but not others [115,120] and 4 detected it for a sub-group of the total population [83,111,113,118]. Moreover, 1 cohort studies have indicated that alcohol use was generally linked to poor cognitive outcomes for the total population [87]. Finally, 3 did not find any significant associations between alcohol consumption and the various cognitive outcomes that were under study [76,77,129]. (See Table 2 and Figure 2A).

9 of the 12 cross-sectional studies (75%) found an association in the hypothesized direction for the entire study population and for most outcomes of interest [106,109,110,119,121,123,126-128]. The remaining 3 studies either found this U-shaped or J-shaped association in a sub-group [107], and either failed to detect any association [112] or detected one that was not in line with the hypothesis, whereby alcohol use was generally found to result in poor cognitive outcomes [130]. (See Table 2 and Figure 2B).

#### Physical activity

Physical activity has many well-known benefits for preventing a number of chronic disorders, including coronary heart disease, stroke, diabetes mellitus and osteoporosis. However, its impact on cognitive functioning has not been studied extensively. Several mechanisms may underlie the potentially protective effects of physical activity on cognitive function, including sustained cerebral blood flow [274], improved aerobic capacity and cerebral nutrient supply [275,276] as well as growth factors, specifically the brain-derived neurotropic factor, which is a molecule that increases neuronal survival, enhances learning, and protects against cognitive decline [277,278].

Currently, 24 cohort and 4 cross-sectional studies have examined the hypothesized relationship. For instance, a recent cohort study of 716 dementia-free older adults from the Rush Memory and Aging Project who were followed-up for an average of 4 years found an inverse relationship between total daily physical activity and incident AD after controlling for age, sex, education, self-report physical, social, and cognitive activities, as well as current level of motor function, depressive symptoms, chronic health conditions, and ApoE4 allele status [156]. Furthermore, a recent cross-sectional study of 9344 women, 65 years and older, found a lower prevalence of cognitive impairment among those who reported being physically active versus those who reported being physically inactive at different stages of their lives [155].

These findings suggested that physical activity could represent an important and potent protective factor for cognitive decline and dementia in elderly persons. Significant findings were obtained by other recent cohort [132-145,147,148,150-154,156-158] and cross-sectional studies [100,146,149]. Only one cohort study resulted in non-significant findings [143].

In sum, of the 24 selected cohort studies linking physical activity to the various cognitive outcomes, 21(87.5%) found the relationship in the hypothesized direction in the entire population that was studied and for most outcomes of interest [132,133,135-140,142,144,145,147,148,150-154,156-158,279], while 1 found this relationship in ApoE4 carriers [134] and 1 in non-carriers [141]. In one cohort study, the association was against its hypothesized direction [143]. (See Table 2 and Figure 2A).

In addition, all 4 of the selected cross-sectional studies (100%) found an association in the hypothesized direction for the entire study population and for most outcomes of interest (See Table 2 and Figure 2B).

### Nutritional factors

Nutritional factors being studied in relation to cognitive outcomes included caffeine consumpion, antioxidant nutrients and Hcy. In addition, special attention was devoted recently to one class of essential fatty acids, namely *n*-3 fatty acids.

#### Caffeine

Caffeine is known to be the most widely used psychoactive drug worldwide. Its main source is coffee particularly in Western diets. Acting as a stimulant of the central nervous system, caffeine causes heightened alertness and arousal [280]. Previous literature yielded inconsistent findings about the effects of caffeine consumption on cognitive processes. In fact, caffeine improved perceptual speed and vigilance, as well as more complex functions such as memory [281]. Caffeine is one type of compound known as methylxanthines whose effects are mainly to block adenosine receptors in the brain, resulting in cholinergic stimulation. It was hypothesized that such stimulation would lead to improved memory [282]. The earliest large cross-sectional study conducted by Jarvis and colleagues found that caffeine improved cognitive performance [159]. Later on, other cross-sectional studies focusing on tea consumption found similar results [100,162,167,170]. Others, however, did not show evidence of a significant protective effect [160,168]. In sum, 4 of 7 selected cross-sectional studies linking caffeine consumption to various cognitive outcomes found the relationship to be in the hypothesized direction in the study population and for most outcomes of interest (57.1%), one found this association in men [100] and two failed to find a significant association [160,168]. (See Table 2 and Figure 2B).

Of 11 cohort studies, positive findings pertained to 3 (27.3%) [163,164,175], though this was found only for coffee intake in two studies [165,169], while 5 recent studies detected this association only among women or for specific exposures [165,169,171-173]. The remaining cohort studies (3 of 11, 27%) did not find an association between caffeine intake and cognitive change [161] or incident dementia [166,174]. Given the paucity of large cohort studies, more research is needed to establish causality (See Table 2 and Figure 2A).

#### Antioxidants: focus on vitamin E

Several findings suggest that oxidative stress may play an important role in the pathogenesis of AD. First, the brains of AD patients have lesions that are associated with exposure to free radicals. Moreover, oxidative stress among these patients is also marked by an increased level of antioxidants in the brain that act as free radical scavengers. Finally, in vitro studies suggest that exogenous antioxidants may reduce the toxicity of β-amyloids in the brains of AD patients [283-285]. Based on these findings, it may be hypothesized that dietary antioxidants may help reduce the risk of AD.

Those epidemiologic studies examined the longitudinal relationship between *supplemental antioxidants* and risk of AD and other dementias found conflicting results: While vitamin C supplement use was related to lower AD risk in one cohort study [178], combined supplementation of vitamin E and vitamin C was associated with reduced prevalence and incidence of AD and cognitive decline in three other cohort studies [189,193,198], whereas another study found this effect to be specific to Vitamin E supplements [186]. These findings of a protective effect of supplemental antioxidant use against cognitive impairment and decline was replicated in a large cohort study [185]. However, there were only borderline or little evidence of a cognitive benefit from use of antioxidant supplements, particularly vitamins C and E, according to at least five independent cohort studies [177,180,187,192,199].

There are several prospective cohort studies on the effect of *dietary antioxidants* on the risk of dementia. One study found that high dietary intake of vitamins C and E may reduce the risk of AD [182] with the relationship most pronounced among smokers. Morris and colleagues [183] found that dietary intake of vitamin E, but not other antioxidants, was associated with a reduced risk of incident AD, although this association was restricted to individuals without the Apolipoprotein E ϵ4 genotype. Similar findings were reported with cognitive decline as an outcome [184]. In a later study when both outcomes were considered it was concluded that certain forms of tocopherols not found in dietary supplements but found only in foods may be at play [194]. This observation was corroborated by at least one recent study [197]. Another study, however, suggested that dietary antioxidants were not able to reduce AD risk [187]. Similarly, Laurin and colleagues [188] found no association between midlife dietary intake of vitamins E and C and dementia incidence. At least five other cohort studies came to a similar conclusion [176,181,201,202]. In addition to examining associations of cognition with vitamins C and E, other studies found that carotenoids, particularly β-carotene intake, may be have beneficial effects of various cognitive outcomes [176], though others were not able to detect such an association [184,201,202].

Irrespective of the source of antioxidants, *plasma concentration* may be a good biomarker for oxidative stress status. In particular, an inverse association between plasma vitamin E among others and poor cognitive outcomes was found in at least two cross-sectional studies [179,190] and two cohort studies [196,200]. Another cross-sectional study, however, did not find evidence of an association between plasma antioxidants, including vitamin E and prevalent AD [191]. In addition, among studies that examined the influence of plasma carotenoids [179,195], only one detected a significant potential protective effect against cognitive impairment [195]. While these results are mixed, they suggest that at least one antioxidant has a protective effect against adverse cognitive outcomes.

In sum, of the 21 selected cohort studies linking antioxidants, with focus on vitamin E, to the various cognitive outcomes, 9 (42.9%) found the relationship to be in the hypothesized direction in the entire population that was studied and for most outcomes of interest [176,178,182,184,189,193,194,197,198], while 5 found this relationship for specific antioxidants or some outcomes but not others [180,183,186,196,200,201] and 1 detected it for a sub-group of the total population [183]. The remaining selected cohort studies (n = 6) did not find a significant association [177,185,187,188,192,199]. (See Table 2 and Figure 2A).

Similarly, of the 6 cross-sectional studies that were selected, 2 (33.3%) found an association in the hypothesized direction for the entire study population and for most outcomes of interest [190,195], 1 found the association to hold only for vitamin E [179], whereas 3 found no significant association [181,191,202]. (See Table 2 and Figure 2B).

#### Homocysteine

An elevated level of plasma concentration of the sulfur amino acid Hcy (hyperhomocysteinemia) is recognized as an independent risk factor for cardiovascular, peripheral vascular, and cerebrovascular disease [286]. Accordingly, a potential influence of hyperhomocysteinemia on cognitive functioning among older adults was postulated and several studies were able to associate high levels of Hcy with increased risk of incident AD or all-cause dementia [206,217,224,228,233,234]. Studies have pointed to selective effect of Hcy on specific domains of cognition [214,287,288]. One explanation could be that Hcy might be affecting certain parts of the brain to a greater extent than others, and studies have linked Hcy to higher degree of white matter hyperintensities and with brain atrophy [289-293].

Even though blood Hcy levels increase with age and diminished renal function, it is largely determined by dietary intake of B-vitamins (mainly B-6 and B-12) and folate which are needed to convert Hcy into methionine and cysteine, through the methylation reactions [294]. Thus, Hcy status in plasma can be modified by dietary interventions. Moreover, vitamin B-12 plasma level has been shown to be inversely related to that of Hcy [295] and studies looking at Hcy levels and cognitive functioning also examined the effect of B-vitamins. In particular, vitamin B-12 was found to be protective against decline in at least three recent studies [204,213,219]. At least five other studies [204,213,214,216,217,219,221] concluded that fotate was protective against cognitive impairment or decline. For Vitamin B-6, two other studies suggested a protective effect [213,219]. An antagonistic interaction of folate and Vitamin B-12 with Hcy’s effect on cognition was noted in other studies [224,296,297]. Aside from its link to cardiovascular disease, Hcy was shown to have neurotoxic and excitotoxic properties *in vitro*[298,299], suggesting a direct influence on cognition.

Overall, of the 19 selected cohort studies linking Hcy to the various cognitive outcomes, 12(63.2%) found the relationship in the hypothesized direction in the entire population that was studied and for most outcomes of interest [206,215,217,219,222-224,228,231,233-235], while 2 found this relationship for some outcomes but not others or a sub-group [213,214] and 5 were not able to detect a significant association [203,209,210,229,230]. (See Table 2 and Figure 2A).

Similarly, of the 14 cross-sectional studies that were selected, 11(78%) found an association in the hypothesized direction for the entire study population and for most outcomes of interest [204,205,207,208,216,218,220,221,225,227,232], 1 found an association only among older adults above age 60y [212], 1 detected it among ApoE4^+^ individuals [226], and 1 found no significant relationship [211]. (See Table 2 and Figure 2B).

#### n-3 fatty acids

Another nutritional factor hypothesized to be protective against cognitive decline is higher intake of *n-*3 fatty acids and/or a better balance of *n*-3/*n*-6 fatty acids. Linoleic(LA ~ 18:2*n*-6) and α-linolenic (LNA ~ 18:3*n*-3) are two types of fatty acids that are essential for all members of the animal kingdom. These fatty acids and their respective derivatives are also commonly referred to as *n*-6 and *n*-3 fatty acids. Their essentiality lies in the fact that they cannot be synthesized *de novo* within the human or animal organism [300].

In the past, *n*-3 fatty acids were classified only as essential because of their ability to alleviate deficiency symptoms that include dermatitis, growth retardation and reproductive failure. However, *n*-3 fatty acids have other important neurological functions, which explain their high concentrations in neural and retinal tissues [301-303]. Some of the longer chain fatty acids that are synthesized from α-linolenic acid include Eicosapentanoic acid (EPA ~ 20:5 *n*-3), which through further elongation, desaturation and β-oxidation produces Docosahexaenoic acid (DHA ~ 22:6 *n*-3). On the other hand, products of linoleic acid which are also termed long-chain *n*-6 fatty acids include gamma-linoleic (GLA ~ 18:3 *n*-6), dihomogammalinolenic acid (DGLA ~ 20:3 *n*-6) and Arachidonic acid (AA ~ 20:4 *n*-6) [304]. Of all organs in the human body (excluding adipose tissue), the nervous system has the highest lipid content. The dry weight of an adult brain is 50% to 60% lipid, and 35% of the lipid content is accounted for by polyunsaturated fatty acids (PUFAs) [305].

A review of scientific articles and biochemistry textbooks [306] suggested that the fatty acid composition of neuronal cell membrane phospholipids reflects their intake in the diet. Fish oils, which contain high levels of C20 and C22 PUFA, exert the most profound influence on brain PUFA concentrations [306]. The ratio between *n*-3 and *n*-6 PUFA may influence various aspects of serotoninergic and catecholaminergic neurotransmission, and it has been shown that by increasing the density of neurotransmitter receptors for acetylcholine and dopamine, dietary *n*-3 PUFA can improve learning and memory processes [307].

Previous observational studies suggested that the biochemical composition of blood components in terms of fatty acids differs significantly between subjects with normal cognitive functioning and patients with some form of cognitive impairment. While the majority of these studies showed an inverse association of plasma and erythrocyte *n*-3 fatty acids with cognition among older adults [243-245,248,255,258], at least one found no association between biochemical markers of *n*-3 fatty acids and cognition [251].

Epidemiological studies involving self-reported dietary data of *n*-3 fatty acids had suggestive but slightly controversial results. One study by Morris and colleagues used cohort data on 815 subjects who were initially unaffected by AD (age range: 65-94y, mean follow-up period = 2.3y). Using standardized criteria, AD incidence was compared across *n*-3 fatty acid consumption groups, with those eating fish once per week compared to those who rarely or never eat fish having considerably lower incidence (RR = 0.4; 95% CI: 0.2, 0.9). Total *n*-3 fatty acid consumption was also associated with a reduced AD risk even after controlling for intake of other dietary fats, vitamin E and for cardiovascular conditions [239]. A similar finding was reported later on for a larger but comparable cohort when looking at fish consumption and cognitive decline over time [241].

In the Zutphen Elderly Study, cognitive functioning and decline over three years were assessed in a cohort of 476 men aged 69-89y using the Mini-Mental State Examination (MMSE). Findings indicated that high linoleic acid intake (the main *n*-6 fatty acid in the diet) was associated with cognitive impairment, even after controlling for age, education, cigarette smoking, alcohol consumption and energy intake (OR = 1.76, 95% CI: 1.04-3.01, comparing highest to lowest tertile). However, there was no distinctive association for *n*-3 fatty acids. Nevertheless, total fish consumption was suggestive of a protective effect, even though it did not reach significance [236].

Another larger cohort study–The Rotterdam Study– recruited 5,386 non-institutionalized participants, aged 55 + y at baseline, who had normal cognition and assessed their complete dietary intake with a semi-quantitative food-frequency questionnaire. After an average 2.1y of follow-up, lower risk of incident dementia and AD was found among fish consumers and therefore among those with higher intake of *n*-3 fatty acids (RR = 0.3; 95% CI: 0.1-0.9) [237]. However, when the study was conducted later with a longer follow-up (mean follow-up period of 6.0 years), it was concluded that high intake of total, saturated, trans fat, cholesterol and low intake of monounsatured fatty acids (MUFA), total PUFA, *n*-6 PUFA and *n*-3 PUFA were not associated with increased risk of dementia or its subtypes [238].

A cross-sectional study of 1,613 subjects aged 45–70 years that examined the association between fatty acid and fish intake with cognitive function, found that the risk of cognitive impairment was reduced with increased consumption of fatty fish and marine *n*-3 PUFA. per Standard Deviation (SD) increased intake, the ORs were 0.81 (95% CI: 0.66, 1.00) and 0.72 (95% CI: 0.57, 0.90), respectively [240]. Another recent study using the Athersclerosis Risk in Communities (ARIC) cohort data suggested that dietary intake of *n*-3 fatty acids (mainly DHA + EPA) reduced the risk of cognitive decline in verbal fluency but not other cognitive domains (i.e. delayed word recall and psychomotor speed). This protective effect was particularly strong among hypertensive subjects [246]. The potentially protective effect of dietary *n*-3 fatty acid was also reported in several other large epidemiological studies [242,247,249,253,254,256-258], but not in others [250-252].

In sum, 7 out of the 18 (39%) selected cohort studies linking *n*-3 fatty acids to the various cognitive outcomes found the relationship in the hypothesized direction in the entire population that was studied and for most outcomes of interest [237,239,247,248,250,253,257], while 1 found this relationship for some outcomes but not others [245], 4 detected it for a sub-group of the total population [242,243,246,258], and 6 found no association [236,238,241,250-252] (See Table 2 and Figure 2A). In addition, all of the 5 (100%) cross-sectional studies that were selected found an association in the hypothesized direction for the entire study population and for most outcomes of interest [240,244,249,254,255] (See Table 2 and Figure 2B).

### Description of study-level characteristics and comparison by risk factor

Table 3 shows descriptive findings of study-level characteristics and compares their distributions across risk factors. Out of the 247 selected studies, 98 were conducted in the US (39.7%), while 104 were carried out in a European country (42.1%), and the remaining 45 studies originated from Asia, Canada and Australia among others (18.2%). The majority of the selected studies were cohort studies (n = 167). Most had only one type of cognitive outcome (72.5%), whereas 24.3% had two, and the remaining 3.2% had 3 or 4 outcomes. 152 studies had confirmed positive findings for most outcomes, exposures and for all study sub-groups (61.5%), while 18.2% (n = 45) had null findings. Partially positive findings were found in around 18.2% while 2% had a finding against the hypothesized direction. Around 40.5% of studies included participants with ages <65y, and the majority had both men and women (84.2%). Incident AD as an outcome was available in 47 studies, while 47 studies included incident dementia as a main outcome of interest. On the other hand, cognitive function as an outcome was found in 83 of included studies, while 62 of those studies had cognitive decline or change as a primary outcome of interest (data not shown). In general, there was an almost even split between studies focusing on cognitive function/decline/change (51.0%) and studies focused on AD/dementia/impairment as outcomes (46.2%). Only 2.8% of the studies examined both categories. When comparing the distribution of those study-level characteristics by risk factor, we found some significant differences for year of publication, country, age group inclusion/exclusion, study design, cognitive outcome type and study finding. In particular, studies on education and cognitive outcomes tended to be published earlier than studies of other risk factors, there were significantly more European studies of n-3 FA compared to other risk factors, while most studies with PA excluded middle aged adults unlike other risk factors. The highest proportion of cohort studies was also found for PA. The vast majority of studies on alcohol and cognitive outcomes used cognitive function/decline as their primary outcome of interest, unlike other risk factors which were more balanced in terms of cognitive outcome type. The percent positive finding was highest among PA studies (89.3%) and lowest for caffeine studies (38.9%). The significant difference in percent “positive finding” was found in cohort studies (p = 0.043) rather than cross-sectional studies (p = 0.09) (See Figure 2A-B).

**Table 3:** Study-level characteristics distribution, overall and comparison across risk factors

	**Overall**		**EDU**		**SMOK**		**ALCO**		**PA**		**CAFF**		**ANTIOX**		**HCY**		**N-FA**		***P*** *
	**N = 247**		**N = 52**		**N = 36**		**N = 30**		**N = 28**		**N = 18**		**N = 27**		**N = 33**		**N = 23**		
Year, Mean (SD)	2004.5	(5.1)	2001.3	(6.3)	2003.6	(5.2)	2004.5	(4.5)	2006.7	(3.6)	2007.7	(4.5)	2003.8	(3.8)	2006.2	(3.1)	2006.9	(4.0)	<0.001
Country, N (%)																			
US	98	(39.7)	16	(30.8)	12	(33.3)	15	(50.0)	17	(63.0)	3	(16.7)	17	(63.0)	13	(39.4)	5	(21.7)	0.020
Europe	104	(42.1)	22	(42.3)	18	(50.0)	9	(30.0)	8	(28.6)	10	(55.6)	8	(29.6)	14	(42.4)	15	(65.2)	
Other	45	(18.2)	14	(26.9)	6	(16.7)	6	(20.0)	3	(10.7)	5	(27.8)	2	(7.4)	6	(18.2)	3	(13.00	
Age group, N (%)																			
Excludes < 65y	147	(59.5)	31	(59.6)	17	(47.2)	13	(43.3)	24	(85.7)	12	(66.7)	20	(74.1)	16	(48.5)	14	(60.9)	0.012
Includes < 65y	100	(40.5)	21	(40.4)	19	(52.8)	17	(56.7)	4	(14.3)	6	(33.3)	7	(25.9)	17	(51.5)	9	(39.1)	
Sex, N (%)																			
Both	208	(84.2)	47	(90.4)	28	(77.8)	28	(93.4)	19	(67.9)	16	(88.9)	22	(81.5)	28	(84.8)	20	(87.0)	0.12
Men only	26	(10.5)	3	(5.8)	8	(22.2)	1	(3.3)	5	(17.9)	2	(11.1)	2	(7.4)	3	(9.1)	2	(8.7)	
Women only	13	(5.3)	2	(3.8)	0	(0.0)	1	(3.3)	4	(14.3)	0	(0.0)	3	(11.1)	2	(6.1)	1	(4.3)	
Study design, N (%)																			
Cross-sectional	80	(32.4)	25	(48.1)	7	(19.4)	12	(40.0)	4	(14.3)	7	(38.9)	6	(22.2)	14	(42.4)	5	(21.7)	0.012
Cohort	167	(67.6)	27	(51.9)	29	(80.6)	18	(60.0)	24	(85.7)	11	(61.1)	21	(77.8)	19	(57.6)	18	(78.3)	
Sample size, Mean (SD)	3,561	(5,128)	4,345	(6,066)	4,745	(6,859)	4,745	(6,808)	3,322	(3,927)	2,408	(2,385)	3,643	(4,191)	1,074	(769)	3,061	(3,389)	0.05
Cognitive outcome count, N (%)																			
1	179	(72.5)	34	(65.4)	26	(86.7)	26	(86.7)	20	(71.4)	14	(77.8)	22	(81.5)	21	(63.6)	15	(65.2)	0.77
2	60	(24.3)	15	(28.8)	4	(13.3)	4	(13.3)	7	(25.0)	3	(16.7)	4	(14.8)	10	(30.3)	8	(34.8)	
3	5	(2.0)	2	(3.9)	0	(0.0)	0	(0.0)	1	(3.6)	1	(5.6)	0	(0.0)	1	(3.0)	0	(0.0)	
4	2	(1.2)	1	(1.9)	0	(0.0)	0	(0.0)	0	(0.0)	0	(0.0)	1	(3.7)	1	(3.0)	0	(0.0)	
Cognitive outcome type, N (%)																			0.042
AD/dementia/impairment	114	(46.2)	26	(50.0)	20	(55.6)	6	(20.0)	16	(57.1)	5	(27.8)	15	(55.6)	15	(45.5)	11	(47.8)	
Cognitive function/decline	126	(51.0)	25	(48.1)	16	(44.4)	24	(80.0)	10	(35.7)	11	(61.1)	12	(44.4)	17	(51.5)	11	(47.8)	
Both	7	(2.8)	1	(1.9)	0	(0.0)	0	(0.0)	2	(7.1)	2	(11.1)	0	(0.0)	1	(3.0)	1	(4.4)	
																			
Study finding, N(%)																			0.004
Against hypothesis	5	(2.0)	1	(1.9)	1	(2.8)	2	(6.7)	1	(3.6)	0	(0.0)	0	(0.0)	0	(0.0)	0	(0.0)	
Null	45	(18.2)	4	(7.7)	11	(30.6)	4	(13.3)	0	(0.0)	5	(27.8)	9	(33.3)	6	(18.2)	6	(26.1)	
Positive	152	(61.5)	39	(75.0)	18	(50.0)	17	(56.7)	25	(89.3)	7	(38.9)	11	(40.7)	23	(69.7)	12	(52.2)	
Partially positive (outcomes/exposures)	21	(8.5)	6	(11.5)	2	(5.6)	2	(6.7)	0	(0.0)	3	(16.7)	2	(7.4)	1	(3.0)	1	(4.3)	
Partially positive (sub-groups)	24	(9.7)	2	(7.7)	4	(11.1)	5	(16.7)	2	(7.1)	3	(16.7)	5	(27.8)	3	(9.1)	4	(17.4)	

*P-value for difference across risk/protective factors was obtained from one-way ANOVA test when variable is continuous and *χ*^2^ test when variable is categorical.

### Consistency analysis: study-level characteristics and risk factor as predictors of study finding

In an attempt to examine heterogeneity in findings across risk factors and study-level characteristics, we conducted a consistency analysis using a logistic regression model (Table 4). Examining the odds ratios and their 95% CI, taking “null finding/against hypothesis finding” as the referent category for the outcome, we found that in general, a positive or partially positive finding was significantly more likely when the risk factor was “education” particularly when compared to smoking, caffeine and antioxidants/vitamin E (p < 0.05). None of the other study-level characteristics were associated with the study finding.

**Table 4:** Multiple logistic regression: study-level predictors of study finding*

	**Odds Ratio**	**(95% CI)**	**P-value**
*Year*	1.06	(0.98;1.14)	0.13
*Country*			
US	1.00		
Europe	0.96	(0.45;2.06)	0.92
Other	0.99	(0.35;2.80)	0.99
*Age group*			
Excludes < 65y	1.00		
Includes < 65y	0.80	(0.40;1.60)	0.53
*Study design*			
Cross-sectional	1.00		
Cohort	0.60	(0.27;1.33)	0.21
*Sample size*	1.00	(0.99;1.00)	0.25
*Cognitive outcome count*	1.03	(0.54;1.98)	0.92
*Cognitive outcome type*			
AD/dementia/impairment	1.00		
Cognitive function/decline	1.52	(0.72;3.22)	0.27
Both	1.78	(0.16;19.7)	0.64
*Risk factor*			
EDU	1.00		
SMOK	0.21	(0.06;0.70)	0.012
ALCO	0.33	(0.08;1.29)	0.11
PA	2.49	(0.26;24.29)	0.43
CAFF	0.18	(0.04;0.84)	0.029
ANTIOX	0.20	(0.06;0.75)	0.016
HCY	0.41	(0.10;1.67)	0.22
N-3 FA	0.25	(0.06;1.05)	0.06

*Positive or partially positive finding coded as “1”, Null or against hypothesized finding coded as “0” (referent category).

Gender composition of the sample was excluded as a predictor due to lack of variability.

### Meta-analysis: selected risk factors for incident AD

**Figure 3:**
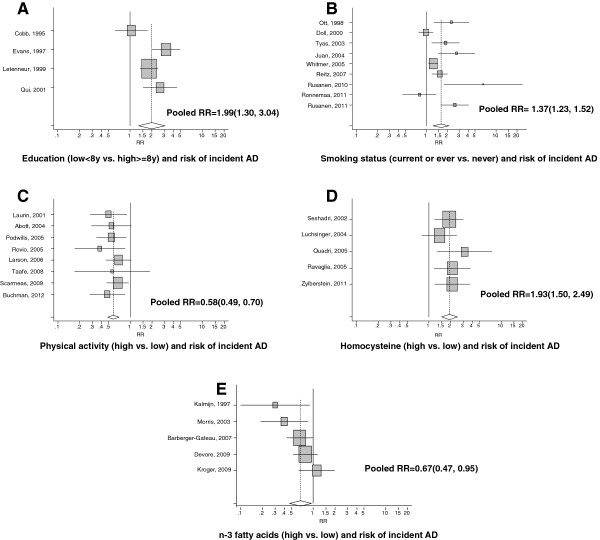
**Meta-analysis of selected risk and protective factors for incident AD (n = 31). (A)** Education. **(B)** Smoking status. **(C)** Physical activity. **(D)** Homocysteine. **(E)** n3 fatty acids.

Using random effects models, we pooled findings of 31 selected data points from 31 studies in which the outcome was incident AD and for which exposure data was adequate and comparable across studies (Figure 3A-E**)**. Among studies used to summarize the association between low education and incident AD, the largest study (N = 3,675) found a HR = 1.81 with 95% CI: 1.30–2.53, with a prevalence of low education being ~32% [43]. In all four studies, the exposure definition was standardized as a comparison between <8y of education vs. ≥8y. In contrast, for all other exposures, definitions differed to some extent between studies but were assumed to operationalize the same concept. For instance, high vs. low physical activity was defined as a frequency of 3 times or more per week by two studies, 2 times of more per week by one, 4 activities per week by one, ≥2 vs. <2 mile walk/day by another study, and other comparable definitions by the remaining four studies combining frequency and intensity of activity. A full description of how various exposures were defined under Figure 3A-E notes.

*Sources:*[28,33,43,46,78,82,86,88,93,95,101,103,104,133,137,141,142,145,148,150,156,206,209,215,217,233,237,239,242,250,251]: *Notes*: Only studies with available data points on incident AD were selected. Moreover, risk factors/protective factors needed to be measured in a comparable manner across studies to allow for estimating a pooled RR with a 95% CI. For education, only four studies out of 27 cohort studies had the required inclusion criteria. For smoking status, the common referent category was non-smoking or never smoking or « never or former » whereas exposed group consisted of either « ever smokers » [78,93], or a pooled value for RR to obtain an approximate « ever smoker » category [86], or current smokers [82,88,95], or mid-life smoking or heavy smoking [101,103,104]. For high vs. low physical activity level, two studies used the cut-point of 3 or more times per week [133,145], one used 2 times or more per week [142], one used the criterion of 4 activities per week vs. none [141], one used > =2 vs. <2 miles walking/day [137], and the remaining four studies used other definitions related to both frequency and intensity [148,150,156]. For high vs. low n-3 fatty acids, one study had fish consumption (yes vs. no) as the exposure of interest [237], another examined quintiles of total *n*-3 PUFA and compared the fifth to the first quintile in terms of risk for AD [239], a third study had one main exposure as « weekly consumption of fish vs. not » [242], a fourth study contrast high *vs*. no fish intake, 0-8y follow-up [250], and finally upper quartile vs. lowest quartile for total n-3 PUFA in erythrocyte membranes [251]. For high vs. low Hcy, two studies used a cut-point of 14.6 μmol/L [206,215], one study used a cut-point of 15 μmol/L [217], one study used upper vs. lowest quartile [209] and one study used upper tertile vs. lowest tertile [233].

Tests of heterogeneity, including the Q-tests, determined whether to use fixed-effects or random-effects models to pool the RR. Findings indicated that, with the exception of education and smoking as main exposures, RR estimates obtained from individual studies were largely homogenous. In sum, the pooled RRs were: 1.99 (1.30, 3.04) for low vs. higher educational attainment (n = 4 data points; Q = 11.33, p = 0.010); 1.37(1.23-1.52) for smoking status (current or ever vs. never smokers (n = 9 data points, Q = 36.2, p < 0.001); 0.58(0.49, 0.70) for physical activity (n = 8 data points, Q = 3.2, p = 0.867), 0.67(0.47,0.96) for high intake of *n*-3 fatty acids (n = 5, Q = 7.4, p = 0.116), and RR = 1.93(1.50, 2.49) for high levels of plasma Hcy (n = 5 data points; Q = 2.64, p = 0.620).

Taking the largest study for each as a means to obtain an estimate of exposure prevalence, the following was found: low education (32%) [43], mid-life smoking (59.8%) [93], *lower* physical activity (62%) [150], *lower n*-3 fatty acids (49.4%) [242], elevated Hcy (30%) [206]. From these exposure prevalence estimates (Prev_exp_), we computed the PAR% and its 95% CI to assess the proportion of AD that is attributable to each exposure in a typical adult population and thus the % that can be averted if that exposure was eliminated from that population. Our findings indicated that the PAR% for low education was 24.0% with a 95% CI: 8.4-39.6; for mid-life smoking it was 31.0% with a 95% CI: 17.9-44.3; for physical activity (lower vs. higher) it was 31.9% with 95% CI: 22.7-41.2; for high vs. low Hcy, it was 21.7% with a 95% CI: 12.8-30.6; for lower vs. higher fish consumption (<weekly vs. ≥weekly), it was 21.9% with 95% CI:4.7-39.1.

Publication bias for the meta-analysis data points (n = 31) was assessed using primarily the funnel plot which plotted point estimates of RR for all exposures combined on the Log_e_ scale against their standard errors. This plot indicated that estimates obtained from those 31 studies lay within the pseudo 95% confidence limits, an indication of non-appreciable publication bias. This finding was reinforced by a non-significant Begg-adjusted rank correlation test (*Z* = 0.25; *P* = 0.80), and by Egger’s regression asymmetry test (bias (SE): -0.43 (0.98); p = 0.66) (Additional file 1: Figure S1).

## Discussion

As stated earlier, this is the first study to systematically review those selected modifiable risk and protective factors for cognitive health outcomes in cross-sectional and cohort studies while comparing the consistency of association between those factors and across study-level characteristics. It is also among few recent studies to compare the strength of association across those factors in relation to incident AD using a similar approach [19,20]. However, our study has a few limitations. First, the literature search was limited to published articles in English available in the Medline database. Second, comparing all included studies in a quantitative meta-analytic manner was not possible due to the diversity of the cognitive outcome measurements between original studies included. In fact, cognitive measures included scores from batteries of different cognitive tests, single global cognitive test scores such as the MMSE total score, as well as the use of a factor analytic approach to combine test scores into various domains of interest (e.g. memory, spatial, psychomotor, executive function, attention). Thus, meta-analysis was only possible for measures of cognitive impairment (i.e. MCI, all-cause dementia, AD, VaD) from which we selected incident AD as the most comparable outcome across studies. However, in order to measure consistency across the selected studies and compare risk factors in terms of consistency, we conducted another type of analysis in which a qualitative outcome of “study finding” was modeled against study-level characteristics. The qualification of a finding as null or positive was based on the main conclusion of each study that was included. This type of analysis does not necessarily discriminate between null findings due to low power, poor quality study *vs*. actual null finding. However, our logistic regression analysis indicated that overall, sample size was not a determining factor for the study finding outcome. Combining findings from meta-analysis and the consistency analyses, we compared evidence level for each risk and protective factor of cognitive health. Other limitations include the lack of comparability in measurements of risk or protective factors in studies with incident AD, which resulted in the exclusion of a few data points in our meta-analysis. However, the datapoints that were included in the meta-analysis were relatively comparable as described in the footnotes of Figure 3A-E. Finally, our study was limited by the inability to create a common quality measure for all studies given the diversity of the exposure variables and the relative importance of having a large sample size given the type of exposure (e.g., a larger sample size is needed for a questionnaire-based exposure *vs*. a blood level based exposure).

Our review shows that over the past several decades many risk or protective factors have been studied in relation to cognitive impairment, dementia (including AD) and cognitive decline. Overall, these studies indicate that modifiable factors including individuals’ socio-economic, behavioral characteristics and dietary intake seem to affect people’s cognitive ability and change over time, as well as the incidence of cognitive impairment, all-cause dementia and AD. It is worth noting, however, that some of the diagnostic criteria for dementia, AD and MCI have changed over time, particularly between the 1990s and the more recent years, as shown in Table 1.

In total, 247 studies were retrieved for systematic review. When conducting consistency analysis for each risk factor/design dyad, we found the % of studies with positive finding, given hypothesis, for most outcomes and study participants to range from ~38.9% for caffeine (27.3 for cohort studies (n = 11), 57.1% for cross-sectional studies(n = 7)) to ~89% for physical activity(87.5% for cohort studies(n = 24); 100% for cross-sectional studies(n = 4)). Consistency analysis confirmed that education-related studies had a significantly higher propensity for a positive or partially positive finding compared to caffeine, smoking and antioxidant-related studies. Meta-analysis of 31 studies with incident AD and selected risk/protective factors yielded pooled RR and 95% CI as follows: RR = 1.99(1.30, 3.04) for low(risk factor) vs. higher education (n = 4 studies; Q = 11.33, p = 0.010); RR = 1.37(1.23, 1.52) for smoking status (current or ever(risk factor) vs. never smokers (n = 9 studies, Q = 36.2, p < 0.001); RR = 0.58(0.49, 0.70) for higher physical activity(protective factor) vs. lower (n = 8 studies, Q = 3.2, p = 0.867), RR = 0.67(0.47,0.96) for higher intake of *n*-3 fatty acids(protective factor) vs. lower (n = 5, Q = 7.4, p = 0.116), and RR = 1.93(1.50, 2.49) for high levels of plasma Hcy(risk factor) vs. lower (n = 6 data points; Q = 2.64, p = 0.620). Given the observed prevalence of exposure from the largest study per risk factor included in each meta-analysis, the population attributable risk percent (PAR%) with its 95% CI was estimated as follows: for low education: 24.0% with a 95% CI: 8.4-39.6; for mid-life smoking it was 31.0% with a 95% CI: 17.9-44.3; for physical activity (lower vs. higher) it was 31.9% with 95% CI: 22.7-41.2; for high vs. low Hcy, it was 21.7% with a 95% CI: 12.8-30.6; for lower vs. higher fish consumption (<weekly vs. ≥weekly), it was 21.9% with 95% CI:4.7-39.1. There was no significant publication bias, taking all selected risk factors for incident AD together.

A large number of epidemiologic studies were initially conducted to examine the effects of socio-economic factors, mainly educational attainment, and were later used to assess the validity of alternative hypotheses regarding the presence of behavioral or health-related mediating factors. To this end, several behavioral and nutritional risk factors were studied in relation to various cognitive outcomes. For instance, it was hypothesized that low SES was associated with higher prevalence of smoking which in turn may affect cognitive performance and change over time. While few studies found weak or no association between smoking and cognitive decline, many others found a positive association whereby smoking increased the risk of decline. Alcohol was found in general to have a U-shaped association with the risk of decline, while caffeine was shown to increase perceptual speed and vigilance as well as memory and other more complex functions in at least two cohort studies and one cross-sectional study. Physical activity was shown to protect against cognitive decline as corroborated by a number of prospective cohort studies.

Among nutritional factors, dietary and supplemental antioxidants were shown in some studies to reduce the risk of cognitive decline while in others they showed no appreciable effect. Other micronutrients including B-Vitamins and folate were shown to be protective against cognitive decline, through their dampening effect on plasma Hcy which was shown to consistently increase the risk of dementia, particularly of the AD type. In addition, studies show that *n*-3 fatty acids with their anti-inflammatory and cardio-protective properties can help reduce the risk of cognitive decline and impairment in some studies but not in others. Among nutritional factors, caffeine seems to be the factor hypothesized to have a protective effect with the smallest number of current studies.

## Conclusions

In conclusion, the consistency of findings between studies varied for each selected risk or protective modifiable factors (highest consistency observed for physical activity). Secondly, a moderate to strong association was observed between some selected factors and incident AD (strongest for low education and elevated Hcy). Combining both criteria (strength of association in the case of incident AD and consistency overall), the strongest evidence thus far is an increased risk with elevated plasma Hcy levels or lower educational attainment and a lowered risk with increased physical activity. Nevertheless, more studies are needed to verify the consistency, particularly regarding caffeine. A comprehensive meta-analysis requires additional research for certain risk factors of incident AD or dementia. For incident AD, selected risk factors may potentially account on average for 21.7%-31.9% of AD cases for each risk factor considered (with 95% CI: 8.4%-44.3%) (highest for mid-life smoking and physical activity), given the estimated prevalence of those factors from the largest available study. Thus, on average, one in five to one in three cases of AD can potentially be averted if those risk factors were eliminated from populations with comparable exposure prevalence.

## Abbreviations

AD: Alzheimer’s disease; VaD: Vascular dementia; DLB: Dementia with Lewy bodies; PD-D: Parkinson’s disease with dementia; MD: Mixed dementia; FTD: Fronot-parietal dementia; OD: Other dementia; MCI: Mild cognitive impairment; ADDTC: Alzheimer's disease diagnostic and treatment centers; DSM-IV: Diagnostic and statistical manual, 4^th^ edition; ICD-10: International classification of disease, 10^th^ edition; NINCDS-ADRDA: National Institute of neurological and communicative disorders and stroke -- the Alzheimer's disease and related disorders association; NINDS-AIREN: National institute of neurological and communicative disorders and stroke--Association Internationale pour la Recherche et l’Enseignement en Neurosciences; ApoE: Apolipoprotein E; PAR%: Population attributable risk percent; OR: Odd ratio; RR: Relative risk; HR: Hazard ratio; CI: Confidence interval; Pr_exp_: Prevalence of each exposure; SE: Standard errors; SD: Standard deviation; Edu: Education; Smok: Smoking status; Alco: Alcohol consumption; PA: Physical activity; CAFF: Caffeine consumption; Antiox: Antioxidants; N-3 Fa: n-3 fatty acids; SES: Socio-economic status; SEP: Socio-economic position; Hcy: Homocysteine; EPA: Eicosapentanoic acid; DHA: Docosahexaenoic acid; DGLA: Dihomogaminalinolenic acid; GLA: Gamma-linoleic acid; AA: Arachidonic acid; PUFA: Polyunsaturated fatty acid; MUFA: monounsaturated fatty acid; MMSE: Mini-mental state examination; B: Both; M: Men; W: Women; UK: United Kingdom; US: United States.

## Competing interests

The authors declare that they have no competing interests.

## Authors’ contributions

MAB: conceptualization, literature search and review, plan of analysis, data management, statistical analysis (including meta-analysis), writing of the manuscript, revision of the manuscript. HAB: Literature search and review, plan of analysis, writing of parts of the manuscript, revision of the manuscript. AG: Literature search and review, plan of analysis, writing of parts of the manuscript, revision of the manuscript. AT: Literature search and reivew, writing of parts of the manuscript, revision of the manuscript. ABZ: Plan of analysis, write-up of parts of the manuscript, revision of the manuscript. YW: Plan of analysis, write-up of parts of the manuscript, revision of the manuscript. All authors read and approved the final manuscript.

## Authors’ information

Youfa Wang and Alan B Zonderman are senior authors.

## Pre-publication history

The pre-publication history for this paper can be accessed here:

http://www.biomedcentral.com/1471-2458/14/643/prepub

## Supplementary Material

Additional file 1: Figure S1 Begg’s funnel plot with pseudo 95% confidence limits. Click here for file
