# Supplementary figures and images for: Epidemiologic studies of modifiable factors associated with cognition and dementia: systematic review and meta-analysis
Source: BMC Public Health. 2014 Jun 24;14:643. doi: 10.1186/1471-2458-14-643 (PMC4099157; doi:10.1186/1471-2458-14-643)

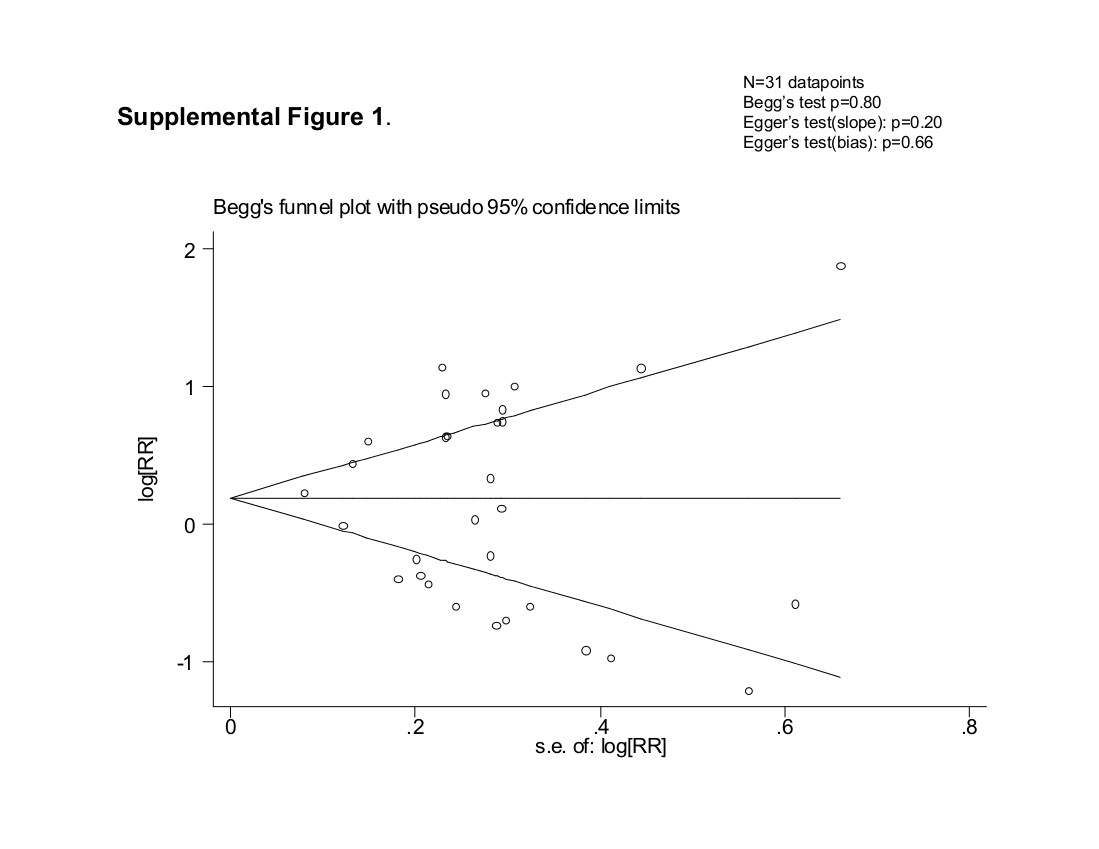

Supplement: Additional file 1: Figure S1 — Begg’s funnel plot with pseudo 95% confidence limits. [file 1471-2458-14-643-S1.jpeg]
